# Orchestrated Biosynthesis of the Secondary Metabolite Cocktails Enables the Producing Fungus to Combat Diverse Bacteria

**DOI:** 10.1128/mbio.01800-22

**Published:** 2022-08-24

**Authors:** Yanlei Sun, Bo Chen, Xinlin Li, Ying Yin, Chengshu Wang

**Affiliations:** a Key Laboratory of Insect Developmental and Evolutionary Biology, CAS Center for Excellence in Molecular Plant Sciences, Shanghai Institute of Plant Physiology and Ecology, Chinese Academy of Sciences, Shanghai, China; b CAS Center for Excellence in Biotic Interactions, University of Chinese Academy of Sciences, Beijing, China; c School of Life Science and Technology, ShanghaiTech University, Shanghai, China; Cornell University

**Keywords:** *Metarhizium*, chemical ecology, master regulator, secondary metabolism, supercluster

## Abstract

Fungal secondary metabolites with antibiotic activities can promote fungal adaptation to diverse environments. Besides the global regulator, individual biosynthetic gene clusters (BGCs) usually contain a pathway-specific transcription factor for the tight regulation of fungal secondary metabolism. Here, we report the chemical biology mediated by a supercluster containing three BGCs in the entomopathogenic fungus Metarhizium *robertsii*. These clusters are jointly controlled by an embedded transcription factor that orchestrates the collective production of four classes of chemicals: ustilaginoidin, indigotide, pseurotin, and hydroxyl-ovalicin. The ustilaginoidin BGC is implicated as a late-acquired cluster in Metarhizium to produce both the bis-naphtho-γ-pyrones and the monomeric naphtho-γ-pyrone glycosides (i.e., indigotides). We found that the biosynthesis of indigotides additionally requires the functions of paired methylglucosylation genes located outside the supercluster. The pseurotin/ovalicin BGCs are blended and mesosyntenically conserved to the intertwined pseurotin/fumagillin BGCs of Aspergillus fumigatus. However, the former have lost a few genes, including a polyketide synthase gene responsible for the production of a pentaene chain used for assembly with ovalicin to form fumagillin, as observed in A. fumigatus. The collective production of chemical cocktails by this supercluster was dispensable for fungal virulence against insects and could enable the fungus to combat different bacteria better than the metabolite(s) produced by an individual BGC could. Thus, our results unveil a novel strategy employed by fungi to manage chemical ecology against diverse bacteria.

## INTRODUCTION

Fungal biosynthetic gene clusters (BGCs) for secondary metabolism are individually regulated by the pathway-specific transcription factor (TF) and/or global regulator(s) (1, 2). There are also examples of a single TF being involved in the control of closely-located or related BGCs. For example, the intertwined fumagillin/pseurotin BGCs are controlled by a Zn(II)_2_Cys_6_-type-TF FapR in Aspergillus fumigatus, which is also epigenetically regulated by the global-regulator LaeA (3). Separate BGCs are involved in the collaborative biosynthesis of polyketide azaphilones in A. terreus, in which three pathway-specific TFs participate in the crosstalk control of metabolite biosynthesis (4). Crosstalk regulation has also been found between two separated BGCs in A. nidulans, in which the overexpression of the TF embedded in one nonribosomal peptide synthetase (NRPS) cluster could activate the TF of a polyketide synthase (PKS) BGC (5). It is still rare to find a single TF involved in the regulation of multiple contiguous or separate BGCs in fungi.

Different secondary metabolites (SMs) produced by fungi have been identified with varied and/or combined activities against bacteria, viruses, and even fungi. For example, the bis-naphtho-γ-pyrone ustilaginoidins produced by the rice pathogen *Ustilaginoidea virens* have antibacterial activities (6). Ustilaginoidin D (UD), as well as its associated aromatic polyketide glycosides (i.e., indigotides), has also been identified in the insect pathogenic fungus Metarhizium *anisopliae* and verified with an effective antitubercular activity against the bacterium Mycobacterium phlei (7). Both fumagillin and pseurotin A (PA) produced by the intertwined BGCs in A. fumigatus have broad antimicrobial activities. The former has been used in epiculture to protect bees against microsporidian infection (8), and the polyketide PA can inhibit both Gram positive (G+) and Gram negative (G–) bacteria (9). Fungal BGCs could be activated after coculturing the fungi with certain bacterial species to induce the production of SM(s) with antibacterial activities (10–12). Thus, the fungal production of antimicrobial SMs may play an essential role in mediating fungal chemical ecology against different microbes. It is anticipated but remains to be investigated whether the collective production of chemicals belonging to different classes could promote the producing fungi to battle diverse bacteria in their environments.

**FIG 1:**
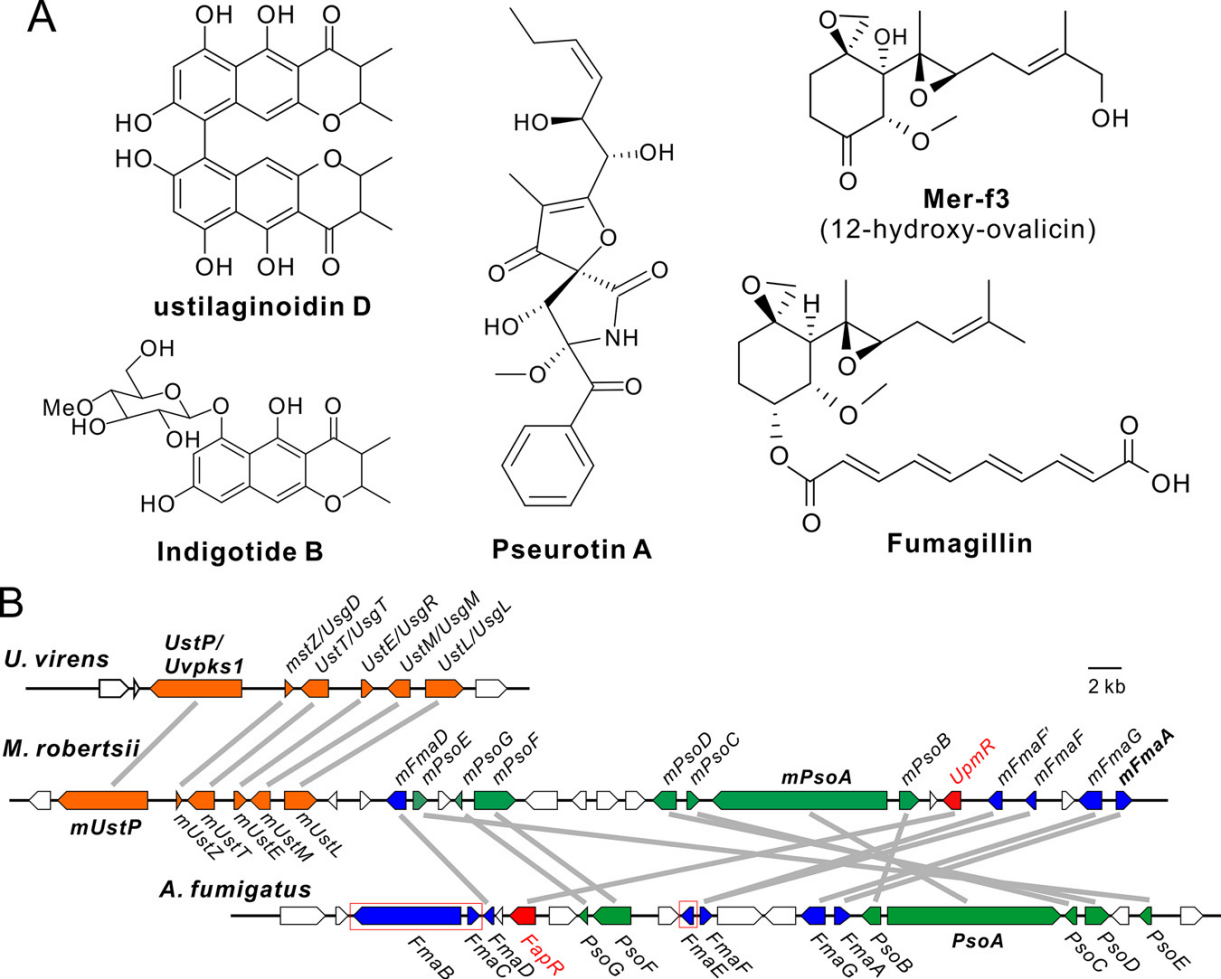
Different chemicals putatively biosynthesized by a supercluster of *M. robertsii*. (A) Different classes of chemicals associated with this study. (B) Comparative structural relationship of the biosynthetic gene clusters encoded in different fungi. The gene models labeled in the same color are putatively involved in the biosynthesis of the same class of compounds. The orthologous genes between fungal species are connected with gray lines. The genes of A. fumigatus framed in red boxes have no homologues in *M. robertsii*. The putative functions of these genes are listed in Table S1.

In this study, we report that *M. robertsii*, with a broad host range against different insects, can produce four classes of compounds: UD, indigotides, pseurotins, and mer-f3 (12-hydroxy-ovalicin) (Fig. 1A). We also report that a supercluster containing three sub-BGCs is responsible for the production of these chemicals (Fig. 1B). It is evident that these sub-BGCs are jointly controlled by a FapR-like TF termed UpmR (as the ustilaginoidin, pseurotin, and mer-f3 biosynthesis regulator). The orchestrated production of chemical cocktails has no obvious contribution to fungal virulence against insect hosts, but it can facilitate the fungus to combat different bacteria in additive and/or synergistic manners.

10.1128/mbio.01800-22.9 TABLE S1 Orthologous relationship of the clustered genes in different fungal species. Download Table S1, PDF file, 0.4 MB. Copyright © 2022 Sun et al. 2022 Sun et al. https://creativecommons.org/licenses/by/4.0/ This content is distributed under the terms of the Creative Commons Attribution 4.0 International license.

## RESULTS

### Co-regulation of the clustered genes within a supercluster by UpmR.

Our genome survey of Metarhizium species identified a supercluster harboring the genes that encode the core enzymes PKS (EXU98524, termed mUstP), hybrid PKS-NRPS (EXU98505, mPsoA), and terpene cyclase (TC, EXU98497, mFmaA), which are present only in the generalist species of Metarhizium with broad host ranges against insects (Fig. S1A; Table S1). The PKS BGC is highly conserved in terms of both similarity and synteny to the UstP/UvPKS1 cluster of *U. virens*. The latter has been characterized for the biosynthesis of ustilaginoidins (13, 14). The remaining clustered genes are conserved with mesosyntenic relationships to the intertwined-cluster members of A. fumigatus for the productions of fumagillin and pseurotin (3). However, the Metarhizium cluster lacks the homologues of the Aspergillus-like PKS *FmaB* and its related genes *FmaC* and *FmaE*, which are jointly required for fumagillin production (Fig. 1B; Fig. S1A). The intertwined cluster of A. fumigatus is regulated by the Zn(II)_2_Cys_6_-type transcription factor (TF) FapR (3). The homologues of this TF are present in the generalist Metarhizium species, such as *M. robertsii* (EXU98502, named UpmR, 36% identity at the amino acid level). The *FapR/UpmR*-like gene is widely absent in fungi, aside from some Metarhizium and Aspergillus species. A phylogenetic analysis revealed that, intriguingly, the Aspergillus homologues diverge into two lineages: those reliably grouped with UpmR and those reliably grouped with FapR (Fig. S1B). Both UpmR and FapR contain a Gal4-like domain, each at their N termini (other proteins may have misannotations). Otherwise, all of these TFs hold three conserved motifs/domains with unknown functions at their C termini (Fig. S1C and D).

10.1128/mbio.01800-22.2 FIG S1 Cluster structuring and the phylogenetic and conservation analyses of the embedded transcription factor. (A) Syntenic relationship of the supercluster or its partial encoded in the selected fungal species. Relative to the supercluster containing three BGCs encoded in Metarhizium species, the ustilaginoidin BGC and the intertwined pseurotin/fumagillin (ovalicin) BGCs are partially distributed in other selected fungal species. The genes labelled in the same color show orthologous relationships. *Therm. thermophilus* represents *Thermothelomyces thermophiles*. (B) Phylogenetic relationship of the UpmR and FapR-like regulators encoded in the selected fungi. The maximum likelihood phylogeny was generated with 500 bootstrap replicates. (C) A MEME analysis identified the presence of three conservative motifs at the C termini of the homologous UpmR-like regulators. (D) The consensus sequences of the three conserved motifs (M1 to M3) identified in panel B. Download FIG S1, TIF file, 2.1 MB. Copyright © 2022 Sun et al. 2022 Sun et al. https://creativecommons.org/licenses/by/4.0/ This content is distributed under the terms of the Creative Commons Attribution 4.0 International license.

**FIG 2:**
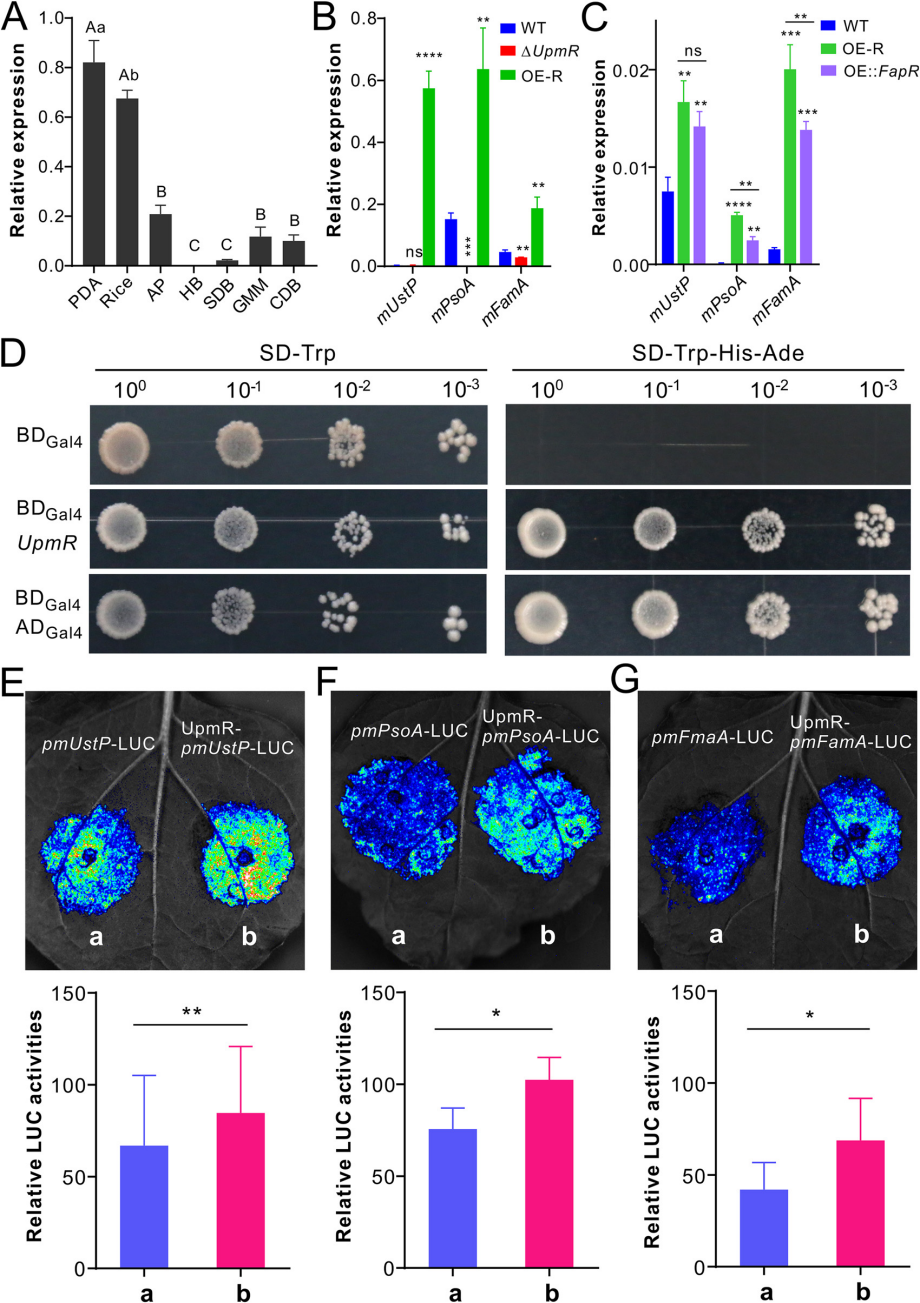
Differential expression, transcription activation, and regulation control of *UpmR* in *M. robertsii*. (A) Differential expression of *UpmR* by *M. robertsii* after being grown in different conditions. OE-R, *UpmR* overexpression mutant. Samples include the conidia harvested from PDA and rice media, appressoria (AP) formed on the fly wings, hyphal body (HB) cells harvested from the wax moth hemolymph, and the mycelia harvested from the SDB, GMM, and CDB broths. Values are presented as mean ± SD. A one-way ANOVA was performed to determine the differences between samples: different capital letters labeled above columns, *P < *0.01; different lowercase letters, *P < *0.05. (B) Differential expressions of three core genes by the WT, *UpmR* deletion, and overexpression (OE-R) strains. (C) Differential expressions of three core genes by the WT, *UpmR*, and *FapR* overexpression mutants. The fungi were grown in GMM for 4 days prior to RNA extraction and gene expression analysis. In panels B and C, values are presented as mean ± SD, and two-tailed Student’s *t* tests were conducted to determine the differences between the WT and the individual mutants: **, *P < *0.01; ***, *P < *0.001; ****, *P < *0.0001; ns, not significant. (D) Confirmation of the UpmR transcription activation feature by yeast two-hybrid analysis. Similar to the positive control (BD_GAL4_-AD_GAL4_), BD_GAL4_-UpmR has an activation activity to enable the yeast cells to grow on the synthetic dropout (SD) medium. Yeast cells transformed with only BD_GAL4_ were used as a negative-control. The serially-diluted yeast cells were grown on SD media for 3 days. (E–G) Targeting of the three core gene promoters by UpmR via dual luciferase (LUC) activity assays in tobacco leaves. Column a represents spots co-infiltrated with the plasmids p1301-35S-Nos and pGreenII-0800-promoter-LUC. Column b represents spots co-infiltrated with the plasmids p1301-35S-UpmR-Nos and pGreenII-0800-promoter-LUC. The lower panels show the corresponding quantitative luminescence intensities (mean ± SD) of each treatment. There were three repeats for each treatment, made on different plants. The differences in signal intensity were compared using two-tailed Student's *t* tests: **, *P < *0.01; *, *P < *0.05.

Since the *UstP*-like cluster is absent in A. fumigatus, we were curious to know whether UpmR is involved in the joint control of this cluster in *M. robertsii*. The expression of *UpmR* was first examined, and it was found that this TF was most highly transcribed by the fungus in conidia harvested from either potato dextrose agar (PDA) or rice media and in the appressorial cells formed on insect wings but not in the hyphal body cells harvested from insect body cavities (Fig. 2A). This TF gene was then either deleted or overexpressed in the wild type (WT) strain of *M. robertsii* (Fig. S2A). After growth on a solid medium, Δ*UpmR* lost the ability of pigmentation, but the overexpression mutant (termed OE-R) did not. The latter, however, had a relatively reduced ability of conidiation (Fig. S2B and C). After growing the fungi in a glucose minimal medium (GMM), it was found that the deletion of *UpmR* could substantially reduce the expression of each core gene, whereas the transcriptions of these genes were significantly increased in the OE-R strain in comparison to those of the WT (Fig. 2B). The other *mUstP-*cluster genes could also be substantially activated by UpmR (Fig. S3A). The Aspergillus *FapR* was also overexpressed in the WT strain of *M. robertsii*, and the three core genes could be similarly upregulated in OE::*FapR*, but this occurred at relatively lower magnitudes than those induced in OE-R (Fig. 2C). Our yeast two-hybrid analysis confirmed that UpmR has a transcriptional activation activity (Fig. 2D). Further, dual luciferase assays confirmed that UpmR could target the promoter of each core gene to activate luciferase activity in tobacco leaves (Fig. 2E). Taken together, UpmR can jointly control three sub-BGCs within this supercluster in *M. robertsii*.

10.1128/mbio.01800-22.3 FIG S2 Verification of gene deletions and phenotyping of different mutants. (A) PCR verification of independent null mutants. *Tub*, a β-tubulin gene, was amplified as a reference control. (B) Growth features of the WT and the different mutants inoculated on GMM agar plates for two weeks. (C) Variation of sporulation between different strains after growth on GMM agar for two weeks. (D) Variation of colony diameter between different strains after growth on GMM agar for two weeks. Panels C and D: the same letter being labelled on two columns indicates no significant difference (*P < *0.05) between the strains after a one-way ANOVA. Download FIG S2, TIF file, 2.1 MB. Copyright © 2022 Sun et al. 2022 Sun et al. https://creativecommons.org/licenses/by/4.0/ This content is distributed under the terms of the Creative Commons Attribution 4.0 International license.

10.1128/mbio.01800-22.4 FIG S3 Gene expression and transcription activation assays. (A) Upregulation of the UD biosynthetic tailoring enzyme genes after the deletion and overexpression (OE-R) of *UpmR*. The WT and *UpmR* mutants were grown in GMM for four days prior to RNA extraction. (B) Induction of the methylglucosylation genes after deletion and overexpression of *UpmR*. The WT and *UpmR* mutants were grown in GMM for four days prior to RNA extraction. (C, D) Dual luciferase (LUC) assays in tobacco leaves, showing that UpmR cannot bind the promoter regions of *MrGT1* and *MrMT1* genes. The lower panels show the corresponding quantitative luminescence intensity (mean ± SD) of each treatment. Column a represents spots co-infiltrated with the plasmids p1301-35S-Nos and pGreenII-0800-promoter-LUC. Column b represents spots co-infiltrated with the plasmids p1301-35S-UpmR-Nos and pGreenII-0800-promoter-LUC. ns, not significant. Download FIG S3, TIF file, 1.6 MB. Copyright © 2022 Sun et al. 2022 Sun et al. https://creativecommons.org/licenses/by/4.0/ This content is distributed under the terms of the Creative Commons Attribution 4.0 International license.

### Production of ustilaginoidin D and indigotides by the *mUstP* sub-cluster.

**FIG 3:**
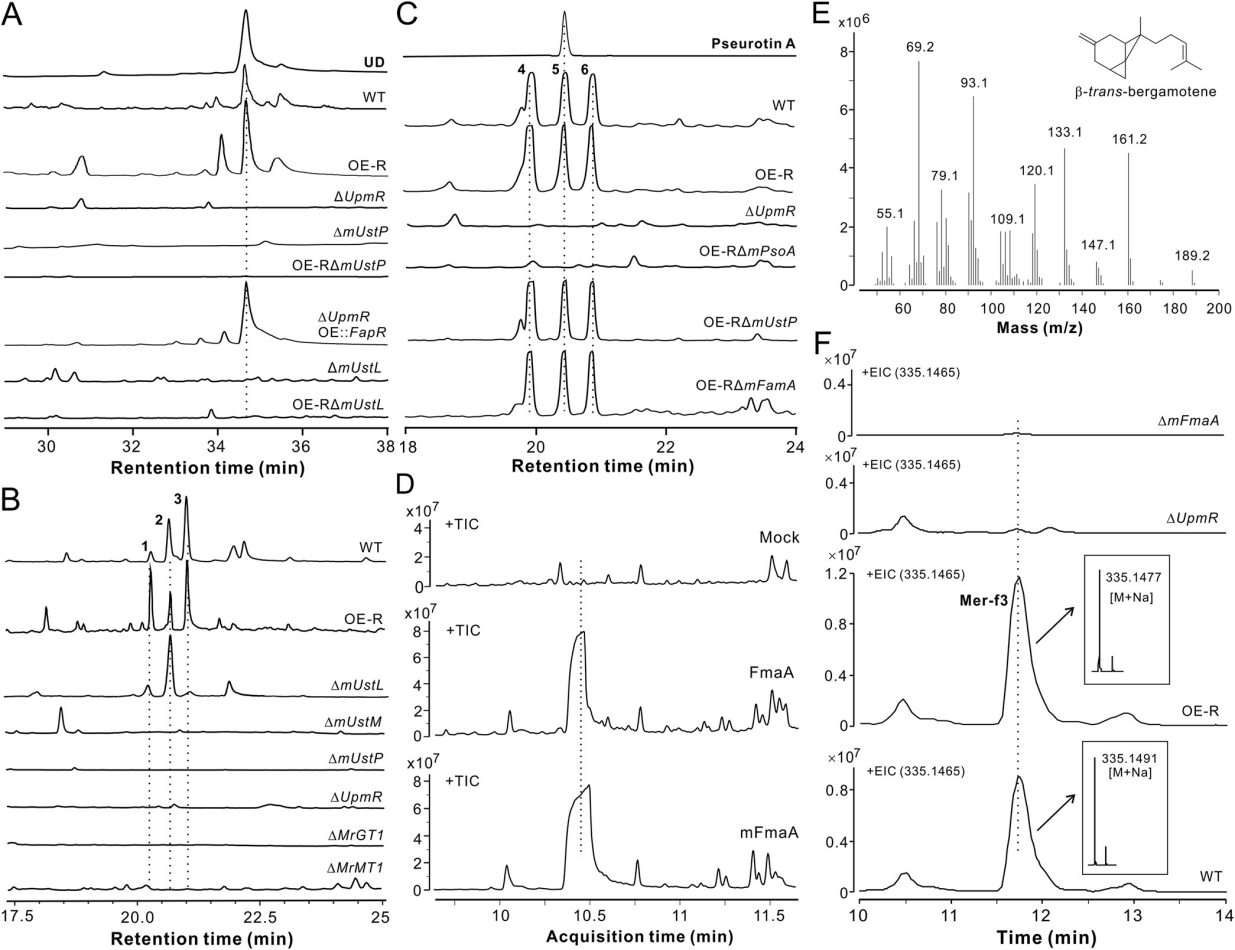
Chemical analysis of the compounds produced by different strains of *M. robertsii*. (A) HPLC profiles showing the production or nonproduction of ustilaginoidin D (UD) by different strains. Purified UD was used as a standard. The fungi were grown in GMM for 7 days, and the mycelia were extracted for analysis. (B) HPLC profiles showing the production or nonproduction of indigotides by different strains. The fungi were grown in GMM broth for 9 days, and the culture filtrates were extracted for analysis. (C) HPLC profiles unveiling the genes involved in the production of pseurotins. Purified pseurotin A was used as a standard. The fungi were grown in GMM for 7 days, and the culture filtrates were extracted for analysis. (D) GC-MS analysis of yeast cells expressing the TC enzymes FmaA of A. fumigatus and mFmaA of *M. robertsii*. Mock yeasts were transformed with an empty vector. TIC, total ion chromatography. (E) Fragmented GC-MS spectra of the compound produced by the yeast cells expressing *mFmaA*. (F) LC-MS profiles confirming the functions of genes for mer-f3 production. The fungi were grown in GMM for 7 days, and the mycelia were harvested and extracted for analysis. EIC, extracted ion chromatography.

Next, we performed the deletion of core genes in the WT and/or OE-R strains and analyzed the compound production profiles of different mutants. After growing the fungi in GMM broth, a high performance liquid chromatography (HPLC) analysis demonstrated that the compounds produced by the WT and OE-R strains disappeared in the Δ*UpmR* and Δ*mUstP* samples (Fig. 3A). The main compound was purified and structurally identified as the known chemical ustilaginoidin D (UD) (Data Set S1). Relative to those produced by *U. virens*, UD is the major ustilaginoidin analog produced by *M. robertsii* (Fig. S4A and B). Consistent with the above gene activation analysis, the overexpression of *FapR* in Δ*UpmR* could restore the ability of the mutant to produce UD. Similarly to the previous analysis of *UstL* function in *U. virens* (13), the deletion of *mUstL* in either the WT or the OE-R strain disabled the mutants’ production of UD (Fig. 3A). Thus, this conserved laccase gene is responsible for the dimerization of monomeric naphtho-γ-pyrones in *M. robertsii*.

10.1128/mbio.01800-22.5 FIG S4 Production of different compounds by the WT and the mutant strains. (A) Parallel comparison of ustilaginoidin production by the WT strains of *U. virens* and *M. robertsii*. UE, ustilaginoidin E; UD, ustilaginoidin D. (B) The mycelial phenotypes of the *U. virens* and *M. robertsii* WT strains after growth in CDB broth for seven days. (C) The production of UD was not affected after the deletions of the other two core genes for pseurotin and mer-f3 biosynthesis in *M. robertsii*. (D) The production of mer-f3 was not affected after the deletions of the other two core genes for UD and pseurotin biosynthesis in *M. robertsii*. Download FIG S4, TIF file, 1.5 MB. Copyright © 2022 Sun et al. 2022 Sun et al. https://creativecommons.org/licenses/by/4.0/ This content is distributed under the terms of the Creative Commons Attribution 4.0 International license.

10.1128/mbio.01800-22.10 DATA SET S1 1D and/or 2D NMR spectra of the compounds identified in this study. Download Data Set S1, PDF file, 0.9 MB. Copyright © 2022 Sun et al. 2022 Sun et al. https://creativecommons.org/licenses/by/4.0/ This content is distributed under the terms of the Creative Commons Attribution 4.0 International license.

Next, we found that the WT, Δ*mUstL*, and OE-R strains could produce three peaks (1 to 3) which were absent in the Δ*mUstP*, Δ*mUstM*, and *ΔUpmR* samples (Fig. 3B). These compounds were purified and structurally identified as indigotides (Data Set S1). In comparison to those reported indigotides (7, 15) (Fig. S5A), peak 2 was identified as the known product indigotide B. Peaks 1 and 3 are new compounds that were named indigotide I and indigotide J, respectively. These metabolites are the methylglucosylated derivatives of monomeric naphtho-γ-pyrones. However, this supercluster does not contain the tailoring enzyme genes of methyltransferase and glucosyltransferase (Table S1). We have recently found the *MrGT1*/*MrMT1* gene pairs in *M. robertsii* that could mediate the sequential glucosylation and methylation of hydroxytenellin produced by Beauveria bassiana during the coculturing of these two fungi (16). We then examined whether MrGT1/MrMT1 would be responsible for the production of indigotides by individual deletions of *MrGT1* and *MrMT1*. As a result, the production of indigotides was abolished in both Δ*MrGT1* and Δ*MrMT1* (Fig. 3B). We also found that both the *MrGT1* and *MrMT1* genes could be highly induced in the OE-R strain (Fig. S3B). However, UpmR did not target the promoters of these two genes (Fig. S3C), suggesting that their expressions might be activated in response to the accumulation of intermediate monomeric naphtho-γ-pyrones.

10.1128/mbio.01800-22.6 FIG S5 Indigotide structures and pseurotin production analysis. (A) The structures of different indigotides reported previously, of which, the indigotides A to F were first identified in the insect pathogenic fungus *Cordyceps indigotica* (anamorph, Metarhizium *indigotica*), while the indigotides G and H were first identified in Metarhizium *anisopliae*. UV-visible absorption (A) and LC-MS (B) analysis of the pseurotin analogs produced by *M. robertsii*. In line with the identified pseurotin A (PA, peak 5 shown in Fig. 3D), peaks 4 and 6 have the same UV-visible spectrum and molecular mass as those of peak 5 (PA), which indicates that these peaks belong to the previously reported analogous PA isomers, pseurotin A_1_ and pseurotin A_2_. Download FIG S5, TIF file, 1.9 MB. Copyright © 2022 Sun et al. 2022 Sun et al. https://creativecommons.org/licenses/by/4.0/ This content is distributed under the terms of the Creative Commons Attribution 4.0 International license.

### Production of pseurotins by the *mPsoA* sub-cluster.

We also found that the WT and OE-R strains could produce three peaks (4 to 6) that were absent in the Δ*UpmR* sample (Fig. 3C). Peak 5 was purified and structurally identified as pseurotin A (Data Set S1). The peaks 4 and 6 had the same the UV-visible spectra and molecular masses as PA (Fig. S5B and C), indicating that they are analogous isomers. Consistent with the finding in A. fumigatus (3), the deletion of the *PsoA* homologue *mPsoA* in the *M. robertsii* OE-R strain abolished the ability of the fungus to produce these pseurotins. Not surprisingly, the null mutants of *mUstP* and *mFmaA* could still produce these compounds (Fig. 3C). Likewise, the deletion of *mPsoA* and *mFmaA* did not affect the production of UD by the fungus (Fig. S4C).

### Production of mer-f3 by the *mFmaA* sub-cluster.

In A. fumigatus, the intertwined *FmaA*-cluster genes are responsible for the production of fumagillin (3). As indicated above, the *FmaA-*like TC gene *mFmaA* (76% identity at the amino acid level with FmaA) is present in *M. robertsii,* but with the absence of the *FmaB*, *FmaC*, and *FmaE* genes, suggesting that Metarhizium would produce an ovalicin-like compound instead of fumagillin, the latter contains a fatty-acid chain. To verify the assumption, we performed the parallel expressions of *FmaA* and *mFmaA* in the yeast strain BY-HZ16, which has been engineered to produce the basal substrate farnesyl-pyrophosphate (FPP) for terpene biosynthesis (17). After yeast fermentations, a gas chromatography-mass spectrometry (GC-MS) analysis identified a product similarly produced by the cells expressing either FmaA or mFmaA (Fig. 3D). A further GC-MS fragmentation analysis obtained the same spectra of the compound produced by FmaA (18), that is, β-trans-bergamotene (Fig. 3E). We then grew the WT and the mutants of *M. robertsii* in GMM, and a LC-MS analysis revealed that the WT and OE-R strains, but not the Δ*mFmaA* and Δ*UpmR* mutants, produced the metabolite with the same extracted ion chromatographic profiles and mass spectra (Fig. 3F). These characteristics belong to the chemical mer-f3 (i.e., 12-hydroxy-ovalicin) that has been previously identified in a Metarhizium species (19). The production of mer-f3 was also not affected in *M. robertsii* after the deletions of the other core genes within the supercluster (Fig. S4D).

### Production of metabolite mixtures enables the fungus to combat different bacteria.

Next, we examined the potential ecology mediated by this supercluster in *M. robertsii*. The *UpmR* and three core gene mutants did not have any obvious defect in forming the infection structure appressoria on hydrophobic surfaces compared with the WT (Fig. S6A). Insect bioassays were then conducted using the WT and the *UpmR* mutant strains for the topical infections of both fruit fly (Drosophila melanogaster) adults and the last instar larvae of wax moth (Galleria mellonella). No obvious difference in insect survival was observed between the WT and the mutants against either the female or the male flies (Fig. S6B and C) or against the moth larvae (Fig. S6D).

10.1128/mbio.01800-22.7 FIG S6 Infection structure induction and insect bioassays of the WT and the mutant strains. (A) No obvious difference in appressorium formation between the WT and the different mutants. Appressorium formation was induced on hydrophobic surfaces for 18 h. AP, appressorium; CO, conidium. Bar, 5 μm. (B, C) Survival of the female (B) and male (C) adults of D. melanogaster after topical infection with the WT and the *UpmR* mutants. The spore suspensions of each strain (1 × 10^6^ conidia/mL) were used, and the controls were treated with 0.05% Tween 20. (D) Survival of the last instar larvae of wax moth (G. mellonella) after topical infection with the WT and the *UpmR* mutants. No statistical difference in fly survival was observed between the WT and the mutants after log-rank tests. Download FIG S6, TIF file, 1.2 MB. Copyright © 2022 Sun et al. 2022 Sun et al. https://creativecommons.org/licenses/by/4.0/ This content is distributed under the terms of the Creative Commons Attribution 4.0 International license.

**FIG 4:**
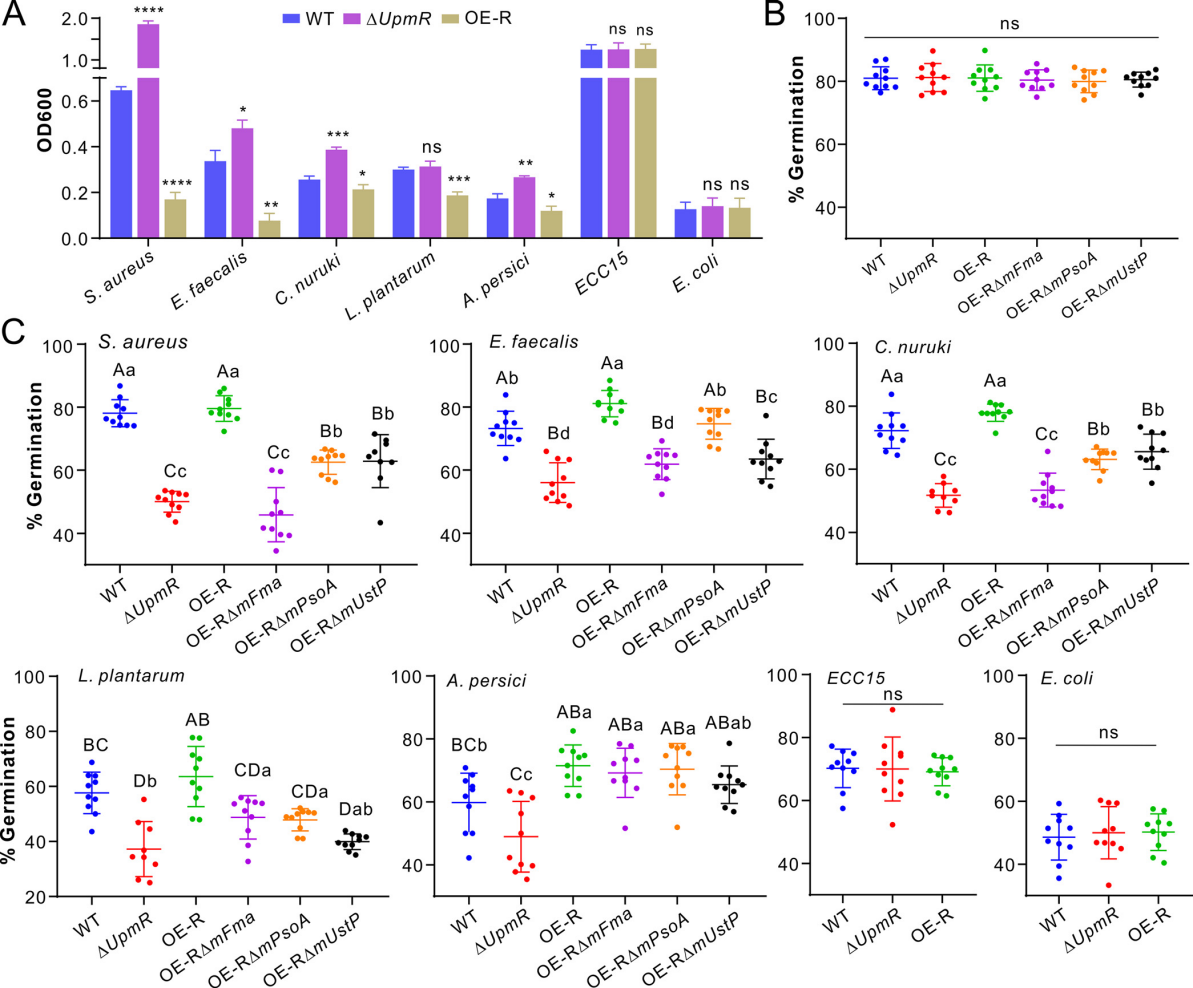
Cross inhibition assays of the WT and mutants of *M. robertsii* versus different bacteria. (A) Varied levels of the inhibition or noninhibition of different bacteria after being cocultured with the WT and mutant strains of *M. robertsii*. The cells of different bacteria were cocultured with fungal spores in LB broth for 12 h, and the bacterial cells were harvested for the measurement of their OD600 values (mean ± SD). There were five replicates for each treatment, and two-tailed Student’s *t* tests were conducted for each bacterial species between the WT and the individual mutants of *M. robertsii*: *, *P < *0.05; **, *P < *0.01; ***, *P < *0.001; ****, *P < *0.0001. (B) No obvious difference in spore germinations between the WT and the different mutants after growth in LB broth for 12 h. (C) Varied levels of spore germinations between the WT and the mutant strains of *M. robertsii* after challenges with different bacteria. The mixed cultures were incubated in LB broth for 12 h. A one-way ANOVA was conducted to determine the differences between strains. Bacterial species are indicated in each panel. Values are presented as mean ± SD. Within each panel, different letters are labeled above: capital letters, *P < *0.01; lowercase letters, *P < *0.05; ns, not significant.

Apart from the infection of insect hosts, *M. robertsii* has to outcompete other microbes for survival and dispersal in the field (16, 20). We found that the deletion or the overexpression of *UpmR* did not apparently impair the ability of the fungus to battle the competitor fungus B. bassiana (Fig. S7A). We then conducted cross inhibition tests between the fungi and different bacteria, and the results showed that the OD600 values of the examined G+ and G– bacteria were largely increased after being cocultured with Δ*UpmR* but substantially reduced after being cocultured with OE-R, compared with the OD600 values of the bacteria grown with the WT strain of *M. robertsii* (Fig. 4A). For example, the OD600 value of Staphylococcus aureus was significantly (*P < *0.0001) increased in the Δ*UpmR* coculture but was significantly reduced (*P < *0.0001) in the OE-R coculture, in reference to the value observed with the WT sample. However, no difference in inhibition was observed between fungal strains against the G– bacteria Erwinia carotovora *carotovora* 15 (*ECC15*) and Escherichia coli. Thus, the production of these chemicals in *M. robertsii* could variably contribute to the fungal inhibition of different G+ bacteria.

10.1128/mbio.01800-22.8 FIG S7 Cross inhibition assays. (A) Fungal competition tests between the different strains of *M. robertsii* and B. bassiana. The spore suspension of the WT strain of B. bassiana (2 μL of 2 × 10^6^ conidia/mL) was inoculated into the middle of the GMM agar plates for three days, and the same volumes of the spore suspensions of the *M. robertsii* WT and the mutants were then inoculated in pairs on both sides for 14 days. No obvious difference in competition was observed between the WT and the individual *UpmR* mutant against B. bassiana. The front and back images of the same Petri dishes were taken and are shown in parallel. (B) Microscopic imaging of the WT and the mutant spore germinations of *M. robertsii* with and without the addition of different bacterial cells. Bar, 5 μm. The spores of the WT and the mutant strains were harvested from two-week old PDA plates. Both the fungal spores (each at a final concentration of 5 × 10^6^ conidial/mL) and the bacterial cells were inoculated in LB broth in test tubes and incubated at 25°C and 220 rpm for 12 h prior to being photographed under a microscope. Download FIG S7, TIF file, 2.1 MB. Copyright © 2022 Sun et al. 2022 Sun et al. https://creativecommons.org/licenses/by/4.0/ This content is distributed under the terms of the Creative Commons Attribution 4.0 International license.

We further tested the bacterial inhibition of fungal spore germinations, and this included the use of core-gene deletion mutants (Fig. S7B). No difference was observed between the WT and the mutants when germinated in the pure nutrient medium (Fig. 4B). In contrast, relative to that of the WT strain, the germination of Δ*UpmR* was significantly reduced, while that of OE-R was either similar or higher after being challenged with the same amount of each G+ bacterium (Fig. 4C). Otherwise, it was interesting to find that there were considerable variations of spore germination among strains. In particular, the germination rates of individual null mutants were considerably lower than that of OE-R when challenged with G+ bacteria. The OE-RΔ*mFmaA* mutant generally had the lowest germination rates, which were similar to those of Δ*UpmR* when challenged with the three G+ bacteria S. aureus, Enterococcus faecalis, and Corynebacterium nuruki, suggesting that mer-f3 would be more important than the other chemicals against these bacteria. Relative to the other strains, the OE-RΔ*mUstP* and Δ*UpmR* had similarly low germination rates in response to the G+ bacterium *Lactiplantibacillus plantarum* (Fig. 4C), suggesting that UD would play a major role in protecting fungal spore germination over the challenge of this bacterium. After being challenged with the G– Acetobacter persici, however, the individual null mutants had similar germination rates to that of OE-R, all of which were higher than that of Δ*UpmR*, suggesting that the combination of any two-cluster compounds could be equally functional in protecting fungal spore germination from the competition of this bacterium. However, there were no differences in spore germination between the WT and the *UpmR* mutants when challenged with *ECC15* and E. coli. In particular, fungal spore germinations could be substantially inhibited by E. coli (Fig. 4C). Overall, the production of chemical mixtures by this supercluster could facilitate *M. robertsii* combating different bacteria, except for some G– bacteria, such as *ECC15* and E. coli, which were examined in this study.

## DISCUSSION

**FIG 5:**
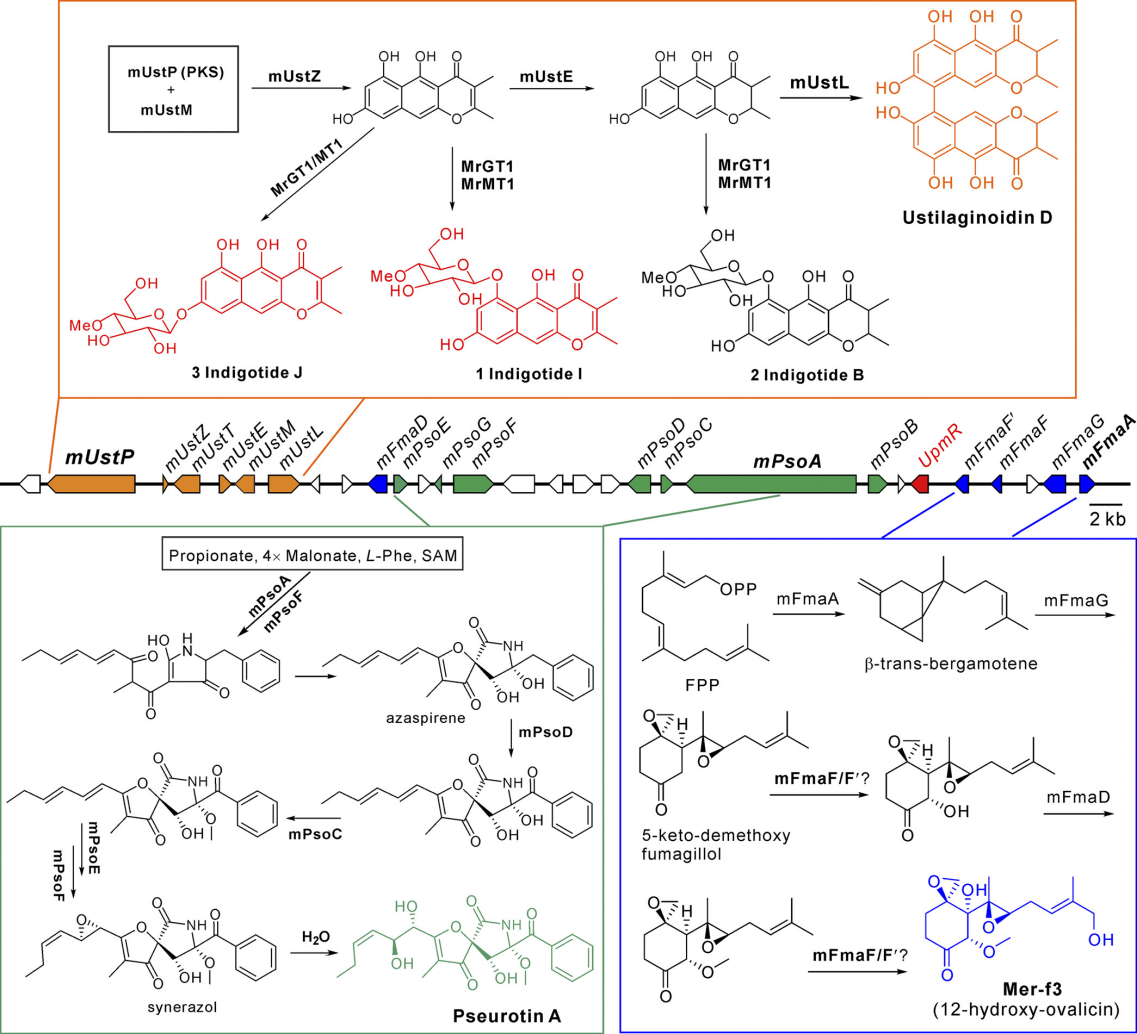
Schematic biosynthesis of the four classes of compounds by the supercluster of *M. robertsii*. The PKS *mUstP* cluster can produce either the pigment ustilaginoidin D or the glycoside indigotides by means of the function of the *MrGT1*/*MrMT1* genes located outside the supercluster. Either 2,3-unsaturated or 2,3-saturated naphtho-γ-pyrone can be used as the substrate of MrGT1/MrMT1. Indigotides I and J are novel compounds identified in this study. FPP, farnesyl-pyrophosphate. SAM, S-adenosyl methionine. The putative function of each gene is listed in Table S1.

In this study, we characterized a secondary metabolic supercluster that is controlled by an embedded regulator to biosynthesize different classes of compounds (Fig. 5). The *mUstP* subcluster is responsible for the biosynthesis of ustilaginoidins, mainly the UD in *M. robertsii*, through the function of PKS mUstP. The catalase mUstL plays a similar function to UstL in *U. virens* to catalyze the formation of the bis-naphtho-γ-pyrones (13, 14). The compound peaks produced by Δ*mUstL* were identified as polyketide glycoside indigotides (IDs), including two new compounds, namely, IDs I and J (Fig. 5). It was interesting to find that these products were catalyzed from the monomeric naphtho-γ-pyrones by MrGT1/MrMT1 tailoring enzyme genes located outside the supercluster (Fig. 5). Thus, in addition to unveiling the UD biosynthesis in Metarhizium, we report for the first time the biosynthetic mechanism of IDs in fungi. In *U. virens*, *UstE* (also called *UsgR*) has been deduced as a Δ^2^-reductase that mediates the reduction of the UstZ product 2,3-unsaturated monomeric naphtho-γ-pyrone to a saturated monomer (13). The IDs B, I, and J were detected in this study, while the IDs G and H have been identified before from *M. anisopliae* (7), a close relative of *M. robertsii*. The identification of these molecules indicates that both the 2,3-unsaturated and saturated naphtho-γ-pyrones can be used as the substrates of MrGT1/MrMT1 (Fig. 5). However, the production ratios of these three IDs varied in the WT, OE-R, and Δ*mUstL* strains (Fig. 3C), indicating a difference in substrate affinity to MrGT1/MrMT1 that remains to be determined. It is worth noting that, consistent with the presence/absence of the *UstP*-like BGC, the orthologues of *MrGT1/MrMT1* are present in *U. virens* but absent in A. fumigatus. Except for the identification of monomeric naphtho-γ-pyrones, such as the hemi-ustilaginoidins D and F from *U. virens* (13), the indigotide-like glycoside has not been reported in this fungus.

The *mPsoA* subcluster contents of *M. robertsii* are conserved to those of the A. fumigatus *PsoA* BGC (3). Thus, a similar mechanism may be involved in the biosynthesis of pseurotin A (Fig. 5). We found that two stereoisomers of PA can be produced by *M. robertsii*, which were deduced to be pseurotins A_1_ and A_2_, with the changes in configuration of C5, C8, and C9 from each other that have also been identified in A. fumigatus (21). Different from the production of fumagillin by A. fumigatus (3), *M. robertsii* produces 12-hydroxy-ovalicin (i.e., mer-f3) through the *mFmaA* subcluster due to its lack of the *FmaB*-like PKS gene for the biosynthesis of a pentaene chain. It was verified that both of the TC enzymes FmaA and mFmaA could similarly catalyze the cyclization of FPP to *trans*-bergamotene. The functions of other enzymes remain to be determined, for example, to verify whether the putative oxygenases mFmaF/F' can mediate the final catalysis of ovalicin to mer-f3.

The homologous counterpart of the *UstP/mUstP* BGCs is absent in A. fumigatus. In the context of evolution, the Metarhizium species are closely related to *U. virens*, and they diverged after this plant pathogen (22, 23). The Clavicipitaceae ancestor of both fungal species is phylogenetically far from A. fumigatus (24). Taken together with the absence of this supercluster in the basal specialist species of Metarhizium (Fig. S1), the highly syntenic relationship between the *UstP* and *mUstp* BGCs would suggest an event of horizontal gene transfer (HGT) in which the Metarhizium generalist species obtained this cluster from *U. virens*. In support, the HGT events of secondary BGCs have been frequently reported between different fungi (1, 25). On the other hand, it cannot be ruled out at this stage that the ancestor of Metarhizium obtained these BGCs from different origins, for example, the ustilaginoidin BGC from *U. virens* and the intertwined PA/fumagillin BGCs from Aspergillus, but the assembled supercluster has been lost in the specialist species during Metarhizium speciation processes. Further support of gene losses in Metarhizium would be the lack of the *FamB*, *FmaC*, and *FmaE*-like genes in the blended PA/ovalicin BGCs of *M. robertsii* compared to that of A. fumigatus. These kinds of HGT and gene gain/loss events reflect a feature of fungal chemical diversity.

Given the highly conserved relationship between the *UstP* and *mUstP* BGCs, we found that *M. robertsii* mainly produced UD, while *U. virens* produced large amounts of UD and UE in the same cultures. More than 20 ustilaginoidin analogs have been identified from *U. virens* (26). It remains to be determined whether *M. robertsii* could similarly produce these structural analogs or evolved divergent control of ustilaginoidin biosynthesis.

We established that the TF UpmR can jointly control these three BGCs within this supercluster, even though the *mUstP* BGC was acquired late by *M. robertsii*. Our BLAST analysis indicated that the *UpmR*-like regulator is absent in *U. virens*. Thus, the control of the *mUstP* cluster would be a functional extension of UpmR. In A. fumigatus, FapR can control only the fumagillin/pseurotin intertwined BGCs and not the fumitremorgin BGC located rather far from the supercluster (3). On the other hand, these three BGCs can be controlled by the global regulator LaeA in A. fumigatus. The homologue of LaeA is also present in *M. robertsii* (EXU95378, 39.4% identity). It remains to be examined whether this methyltransferase is similarly involved in the epigenetic control of this supercluster on top of UpmR in Metarhizium species. It is noteworthy that the overexpression of *FapR* in *M. robertsii* could similarly upregulate the expression of three core genes but that this occurred with reduced magnitudes compared to the effect of *UpmR* overexpression. Apart from the sequence divergence between these two TFs, the variation in binding motifs and/or binding affinity requires further investigation.

We found that the *UpmR* supercluster is dispensable for the virulence of *M. robertsii* against insect hosts. Fumagillin has been found to have tissue damaging activity and to therefore contribute to invasive aspergillosis (27). The related product mer-f3 of *M. robertsii* was found with a similar immunosuppressive activity to the drug cyclosporine against lymphocyte cells (19). The production of cyclosporine was found with a contribution to the virulence of *Tolypocladium inflatum* against insects (28). The similar virulence results of the WT, Δ*UpmR*, and OE-R strains would suggest that the production of mer-f3 had no contribution to the pathogenicity of *M. robertsii*. The direct injection of pseurotin A into wax moth larvae could induce a dose-dependent and reversible paralysis (i.e., one without an insecticidal activity) (29). We found that *UpmR* was not transcribed in the hyphal body cells harvested from insect body cavities. These observations suggest that the supercluster-oriented chemicals would not be produced by *M. robertsii* during the fungal colonization of insect hosts and are therefore nonessential for fungal pathogenicity.

The chemical biology or ecology of fungal secondary metabolisms has been publicly recognized (2, 30). However, the product(s) of a single BGC have usually been investigated. For example, the biosynthesis of cyclodepsipeptide destruxins is required for the virulence of Metarhizium by the evasion of insect immune responses (31). The bis-naphthopyrone aurofusarin produced by Fusarium and the xanthones produced by A. nidulans can protect the producing fungi from a wide range of invertebrate predators (32, 33). The bibenzoquinone oosporein produced by B. bassiana could assist with the fungal infection of insects and limit the *in insecta* bacterial growth after the fungal killing of the insects (34, 35). It was found in this study that the four classes of chemicals produced by the UpmR BGC in *M. robertsii* function as a cocktail that defends the fungus against diverse bacteria or against a single bacterial species with a higher efficiency than what could be provided by an individual BGC. Considering that *UpmR* is highly transcribed by *M. robertsii* in conidia, the production of these chemicals may serve to benefit fungal adaptation to diverse niches containing distinctive bacteria. Apart from additive effects, synergistic effects of chemical cocktails can also be expected, as the use of multiple antibiotics has shown promising effects in combination therapies to treat bacterial infections, especially against drug-resistant bacteria (36).

In conclusion, we report the collective production of four classes of chemicals by a supercluster containing three BGCs in *M. robertsii*. The unclustered *MrGT1/MrMT1* genes were found to catalyze the production of monomeric naphtho-γ-pyrone glycosides, the first mechanism of indigotide biosynthesis. In addition to reporting the coopted control of fungal secondary metabolism, our results advocate a “the-more-the-better strategy” for managing effective chemical ecology in fungi through the production of metabolite cocktails.

## MATERIALS AND METHODS

### Microbial strains and maintenance.

The WT strain ARSEF 2575 of *M. robertsii* and Af293 of A. fumigatus were used in this study. The WT and mutant strains were maintained on potato dextrose agar (PDA, BD Difco) at 25°C. Both the WT and mutant strains of *M. robertsii* were also grown on a rice medium for metabolite isolation. Fungi were grown in liquid media, including Sabouraud dextrose broth (SDB, BD Difco), glucose minimal medium (GMM, NaNO_3_, 6 g/L; KCl, 0.52 g/L; MgSO_4_·7H_2_O, 0.52 g/L; KH_2_PO_4_, 1.52 g/L; glucose, 10 g/L; plus 1 mL/L of a trace element solution) (3), and Czapek-Dox broth (CDB, BD Difco). Appressoria were induced on hydrophobic surfaces of Petri dishes containing the minimum medium plus 1% (vol/vol) glycerol (37). Luria-Bertani (LB) broth (Thermo Fisher Scientific) was used to grow different bacteria and/or fungi. The yeast (Saccharomyces cerevisiae) strain BY-HZ16 (Genotype: *rox1*Δ;*erg9*Δ;*yjl064w*Δ;*ypl062w*Δ) was used and grown in yeast peptone dextrose (YPD, BD Difco) medium for the expression of terpene cyclase (17). The yeast strain Y2H-Gold was used for the two-hybrid analysis using synthetic drop-out (SD) media (Clontech). The bacterial species used for the cross inhibition assays include those isolated from the body surfaces of D. melanogaster (i.e., *L. plantarum* [G+], *A. persici* [G–], *C. nuruki* [G+], and E. faecalis [G+]) (38), and those from the common species of S. aureus (G+), E. carotovora *carotovora* 15 (G–), and E. coli (G–).

### Bioinformatic and phylogenetic analysis.

A whole-genome analysis of secondary metabolic gene clusters was conducted for different Metarhizium species and for other fungi using the program antiSMASH (ver. 6.0) (39). The homologs of UpmR and FapR were retrieved from the NCBI database via a BLASTP analysis. Motif scanning of these UpmR/FapR-like TFs was performed using the MEME (Multiple Expectation maximizations for Motif Elicitation) Suite (ver. 5.4.1) tools (40). The phylogenetic relationships of these selected TFs were inferred using the maximum likelihood method with 500 bootstrap replicates and a Dafhoff model for substitution with the program MEGA X (41).

### Genetic modification and fungal transformation.

To examine the functions of the core genes and the tailoring enzymatic genes encoded by the supercluster (Table S1), we performed individual gene deletions by homologous recombination via *Agrobacterium*-mediated transformation of the *M. robertsii* WT strain. The *MrGT1* and *MrMT1* genes located outside the supercluster were also deleted. In brief, the 5′-flanking and 3′-flanking regions of each target were amplified with different primer pairs (Data Set S2) and cloned into the binary vector pDHt-SK-Bar (42). A drug-resistant colony was selected on medium containing ammonium glufosinate at a final concentration of 200 μg/mL and was verified by a polymerase chain reaction (PCR) and a reverse transcription polymerase chain reaction (RT-PCR) analysis. In addition, the TF *UpmR* was cloned into the binary vector under the control of the constitutive promoter *Tef* for the transformation of the WT strain to obtain the overexpression mutant OE-R (43). For gene deletion in the OE-R strain, the flanking regions of the target gene were cloned into the binary plasmid pDHt-SK-Sur (containing the resistance gene *Sur* against sulfonylurea) for fungal transformations. The full open reading frame of the A. fumigatus TF *FapR* was also cloned and overexpressed in the Δ*UpmR* strain of *M. robertsii* to obtain the mutant Δ*UpmR*OE::*FapR* for the examination of the heterologous complementation of Δ*UpmR* in the control of metabolite production.

10.1128/mbio.01800-22.11 DATA SET S2 The PCR primers used in this study. Download Data Set S2, XLSX file, 0.2 MB. Copyright © 2022 Sun et al. 2022 Sun et al. https://creativecommons.org/licenses/by/4.0/ This content is distributed under the terms of the Creative Commons Attribution 4.0 International license.

### Quantitative gene expression analysis.

The WT, *UpmR* deletion, and overexpression mutants were grown in different media for a quantitative PCR (qPCR) analysis of different genes. The strains were inoculated on PDA and rice medium for 2 weeks to harvest conidial spores for RNA extraction with the TRIzol reagent. The appressorial cells induced on fly wings for 18 h and the hyphal body cells harvested from wax moth larvae 3 days post injection with fungal spores were also used for RNA extraction. Otherwise, the WT and mutant strains were grown in SDB, GMM, and CDB liquid media for 4 days, and the mycelia were harvested by filtration and washed thrice with sterile water prior to RNA extraction. The conversion of RNA to cDNA was conducted using the ReverTra Ace qPCR RT kit (Toyobo, Japan), and a qRT-PCR analysis was performed using different primer pairs (Data Set S2) and the Hieff qPCR SYBR green Master Mix (Yeasen, Shanghai). A β-tubulin gene of *M. robertsii* was used as an internal reference (44).

### Transcription activation assays of UpmR.

We performed transcription activation tests of UpmR in both yeast cells and tobacco leaves. The cDNA of *UpmR* was cloned into the NdeI and BamHI restriction sites of vector pGBKT7 under the control of the GAL4 promoter. For a positive-control, the activation domain (AD) of GAL4 was also cloned from the plasmid pGADT7 and made in tandem with the GAL4 DNA binding domain. The pGBKT7 without an AD domain was used for transformation of the yeast cells AH109 as a negative-control (45). For the transformation of the tobacco leaves, the promoter region (approximately 1.5 kb upstream of the start codon) of the three core genes *mUstP*, *mPsoA*, and *mFmaA* were individually cloned into the vector pGreenII-0800 to control the luciferase gene. The cDNA of *UpmR* was cloned into the vector p1301-35S-Nos (control vector) to obtain the plasmid p1310-35S:UpmR-Nos. The obtained vectors were cotransformed into the *Agrobacterium* strain GV3101 for infiltration of the 3-week-old tobacco (Nicotiana benthamiana) leaves for a dual luciferase (Luc) assay (46). Each treatment was repeated three times on different plants. The treated plants were incubated for two additional days prior to infiltration with 1 mM luciferin. The fluorescent Luc signal was recorded with a Chemiluminescent Imaging System Tanon 5200 (Biotanon, Shanghai).

### Chromatography and mass spectrometry (MS) analysis.

After growing the WT and the mutant strains on a rice medium for 2 weeks or in GMM or CDB liquid media for 9 days, the mycelia and/or culture filtrates were extracted with ethyl acetate. After concentration by rotary evaporation, the samples were re-dissolved in methanol and subjected to a HPLC analysis using the LC-20 AD system (Shimadzu, Japan) equipped with a C18 column (Athena, particle size of 5 μm, 4.6 × 250 mm) and a diode-array detector monitored at 254 nm (16). Eluates were maintained at a flow rate of 1 mL/min with deionized water and acetonitrile (0 to 5 min: 15% CH_3_CN; 5 to 35 min: 15% to 100% CH_3_CN; 35 to 40 min: 100% CH_3_CN; 40 to 45 min: 100% to 15% CH_3_CN; and 45 to 50 min: 15% CH_3_CN). The purification of UD, PA, and indigotides was performed using a semi-preparative C18 reverse-phase column (particle size of 5 μm, 10 × 250 mm; Athena, China), and the samples were eluted at a flow rate of 3 mL/min.

A high-resolution electrospray ionization mass spectrometry analysis of the purified compounds was performed using an Agilent QTOF 6545 instrument operated in the positive ion mode (16). The mycelial extracts of the WT, OE-R, Δ*UpmR*, and Δ*mFmaA* from the GMM medium were analyzed to examine the production or nonproduction of mer-f3 via a GC-MS analysis with the Agilent 5977B system. The 1D or 2D nuclear magnetic resonance (NMR) spectrum data were recorded for each purified compound using a Bruker Avance III-500 spectrometer. Chemical shift values (*δ*) are given in parts per million (ppm), and the coupling constants (*J* values) are given in Hz. Chemical shifts were referenced to the residual solvent peaks of pyridine-*d*5 for the UD and the indigotides, and CD_3_OD-*d*_4_ for the PA compound.

### Heterologous expression of the terpene cyclase and GC-MS analysis.

We expressed the TC *mFmaA* gene in parallel with the Aspergillus *FmaA* (18) in the yeast strain BY-HZ16 as previously described (17). The cDNA of *mFmaA* was amplified with the primers mFmaAF and mFmaAR (Data Set S2) and cloned into the restriction sites *Nde* I/*Pme* I of vector pRS425m. The A. fumigatus *FmaA* was amplified using the primers FmaAF/FmaAR and cloned into the *Nde* I/*Pme* I restriction sites of the pRS425m plasmid. The obtained vectors were individually transformed into the yeast BY-HZ16 cells. The positive clones were grown in YPD broth for 5 days at 28°C and 230 rpm. The yeast cells were harvested by centrifugation, washed twice with sterile water, and then broken by ultrasonication. The samples were extracted twice with hexane for a GC-MS analysis with the Agilent 5977B system (17).

### Insect bioassays.

The topical infection assays were conducted against the male and female adults of D. melanogaster W1118 and the last instar larvae of wax moths (G. mellonella) (43). The conidia of the WT, Δ*UpmR*, and OE::*UpmR* mutants were harvested from 2-week-old PDA plates and suspended in 0.05% Tween 20. The concentrations of spore suspensions were adjusted to 1 × 10^6^ conidia/mL for topical infections of either male or female flies by immersion for 30 sec. There were more than 70 flies used for each treatment, and the experiments were repeated twice. The *Galleria* larvae were immersed in spore suspensions of 2 × 10^7^ conidia/mL. There were more than 45 larvae used for each treatment, and the experiments were repeated twice. Insect mortalities were recorded every 12 h, and the insect survival curves were generated and compared via a Kaplan-Meier analysis using the log-rank test (42).

### Cross inhibition tests.

The WT, Δ*UpmR*, OE-R, and core-gene null mutants were used for cross competition and inhibition assays against B. bassiana and different bacterial species. The natural competitor B. bassiana ARSEF 2860 strain was used for competition tests with the WT and the *UpmR* mutants by pairing tests (28). The spore suspension of B. bassiana (2 μL of 2 × 10^6^ conidia/mL) was first inoculated into the middle of GMM agar plates (9 cm in diameter) for 3 days. The same volumes of the spore suspensions (each at 2 × 10^6^ conidia/mL) of Metarhizium WT, Δ*UpmR*, and OE-R were then inoculated in pairs on both sides of the B. bassiana colonies for 2 weeks to examine the competition between the B. bassiana and the Metarhizium strains. For the cross inhibition assays, the spore suspensions of the Metarhizium WT and the mutant strains (each at a final concentration of 2 × 10^6^ conidia/mL) were cocultured with different amounts of bacterial cells (at a final OD600 = 0.01 for S. aureus; OD600 = 0.05 for *L. plantarum* and *A. persici*; OD600 = 0.005 for E. faecalis and *C. nuruki*; and OD600 = 0.0001 for E. coli and *ECC15* after trial assays) in 50 mL flasks, each containing 20 mL of LB broth at 25°C and 220 rpm for 12 h. The flasks were stood for 5 min for fungal cells to sink, and the OD600 values of bacterial cells were measured with a BioPhotometer (Eppendorf). Two-tailed Student's *t* tests were conducted to compare the differences between the WT and the *UpmR* mutants against each bacterial species. There were three replicates conducted for each paired sample. The spore germinations of the WT and the mutants were examined under a microscope, and the rates of spore germination were determined from the microscopic fields. The counts from every 10 fields were used as a repeat, and there were at least 10 replicates for each sample. A one-way ANOVA was conducted to determine the differences in germination rates between the WT and the individual mutants of *M. robertsii* after challenges with different bacterial species.
