# Supplementary material for: Orchestrated Biosynthesis of the Secondary Metabolite Cocktails Enables the Producing Fungus to Combat Diverse Bacteria
Source: mBio. 2022 Aug 24;13(5):e01800-22. doi: 10.1128/mbio.01800-22 (PMC9600275; doi:10.1128/mbio.01800-22)
Supplement: TABLE S1 [file mbio.01800-22-s0008.pdf]

Table S1. Orthologous relationship of the clustered genes in different fungal species.

M. robertsii	Gene	Annotation	*U. virens*	*A. fumigatus*
EXV01523	MrGT1	UDP-glucosyltransferase	UV8b_01806	/
EXV01522	MrMT1	FkbM family methyltransferase	UV8b_01807	/
EXU98528		hypothetical protein	UV8b_03447	/
EXU98527		amino acid permease	UV8b_05995	AfuA_5g00710
EXU98526		oxidoreductase	UV8b_05851	/
EXU98525		hypothetical protein	UV8b_03468	AfuA_3g00850
**EXU98524**	***mUstP***	**polyketide synthase**	**UV8b_01134, UstP/ UvPKS1**	**/**
EXU98523	*mUstZ*	EthD domain-containing dehydratase	UV8b_01135, UstZ/UsgD	/
EXU98522	*mUstT*	Major facilitator superfamily transporter	UV8b_01136, UstT/UsgT	/
EXU98521	*mUstE*	Phospholipid methyltransferase	UV8b_01137, UstE/UsgR	/
EXU98520	*mUstM*	Methyltransferase	UV8b_01138, UstM/UsgM	/
EXU98519	*mUstL*	Laccase	UV8b_01139, UstL/UsgL	/
EXU98518		IDI-3 protein	/	/
EXU98517		hypothetical protein	/	/
/	/	polyketide synthase	/	AfuA_8g00370, FmaB
/	/	DUF1100 domain protein	/	AfuA_8g00380, FmaC
EXU98516	*mFmaD*	O-methyltransferase B	/	AfuA_8G00390, FmaD
EXU98515	*mPsoE*	Glutathione S-transferase	/	AfuA_8g00580, PsoE
EXU98514		hypothetical protein	/	AfuA_8g00550
EXU98513	*mPsoG*	hypothetical protein	/	AfuA_8g00450, PsoG
EXU98512	*mPsoF*	steroid monooxygenase	/	AfuA_8g00440, PsoF
EXU98511		propionyl-CoA synthase	/	AfuA_8g00500
EXU98510		methionine aminopeptidase 1	/	AfuA_8g00460
EXU98509		methionine aminopeptidase 2	/	AfuA_8g00410
EXU98508		F-box domain, cyclin-like protein	/	AfuA_8g00570
EXU98507	*mPsoD*	Cytochrome P450 CYP5058A4	/	AfuA_8g00560, PsoD
EXU98506	*mPsoC*	methyltransferase	/	AfuA_8g00550, PsoC
**EXU98505**	***mPsoA***	**Hybrid PKS-NRPS**	**/**	**AfuA_8g00540, PsoA**
EXU98504	*mPsoB*	alpha/beta hydrolase	/	AfuA_8g00530, PsoB
EXU98503		Dimeric alpha-beta barrel	/	AfuA_8g00430
**EXU98502**	***UpmR***	**C6 finger transcription factor**	**/**	**AfuA_8g00420, FapR**
/	/	DUF4188 domain protein	/	AfuA_8g00470, FmaE
EXU98501		2OG-Fe(II) oxygenase	/	AfuA_8g00480, FmaF
EXU98500	*mFmaF*	2OG-Fe(II) oxygenase	/	
EXU98499		hypothetical protein	/	
EXU98498	*mFmaG*	Cytochrome P450 CYP620F3	/	AfuA_8g00510, FmaG
EXU98497	***mFmaA***	**Terpene cyclase**	/	**AfuA_8g00520, FmaA**
EXU98496		FAD-binding protein	/	

Note: MrGT/mrMT gene pairs are shadowed that are located outside the supercluster; Core genes are highlighted in bold. / shows the absence of the homologous genes in respective fungal species.
