# Supplementary figures and images for: Orchestrated Biosynthesis of the Secondary Metabolite Cocktails Enables the Producing Fungus to Combat Diverse Bacteria
Source: mBio. 2022 Aug 24;13(5):e01800-22. doi: 10.1128/mbio.01800-22 (PMC9600275; doi:10.1128/mbio.01800-22)

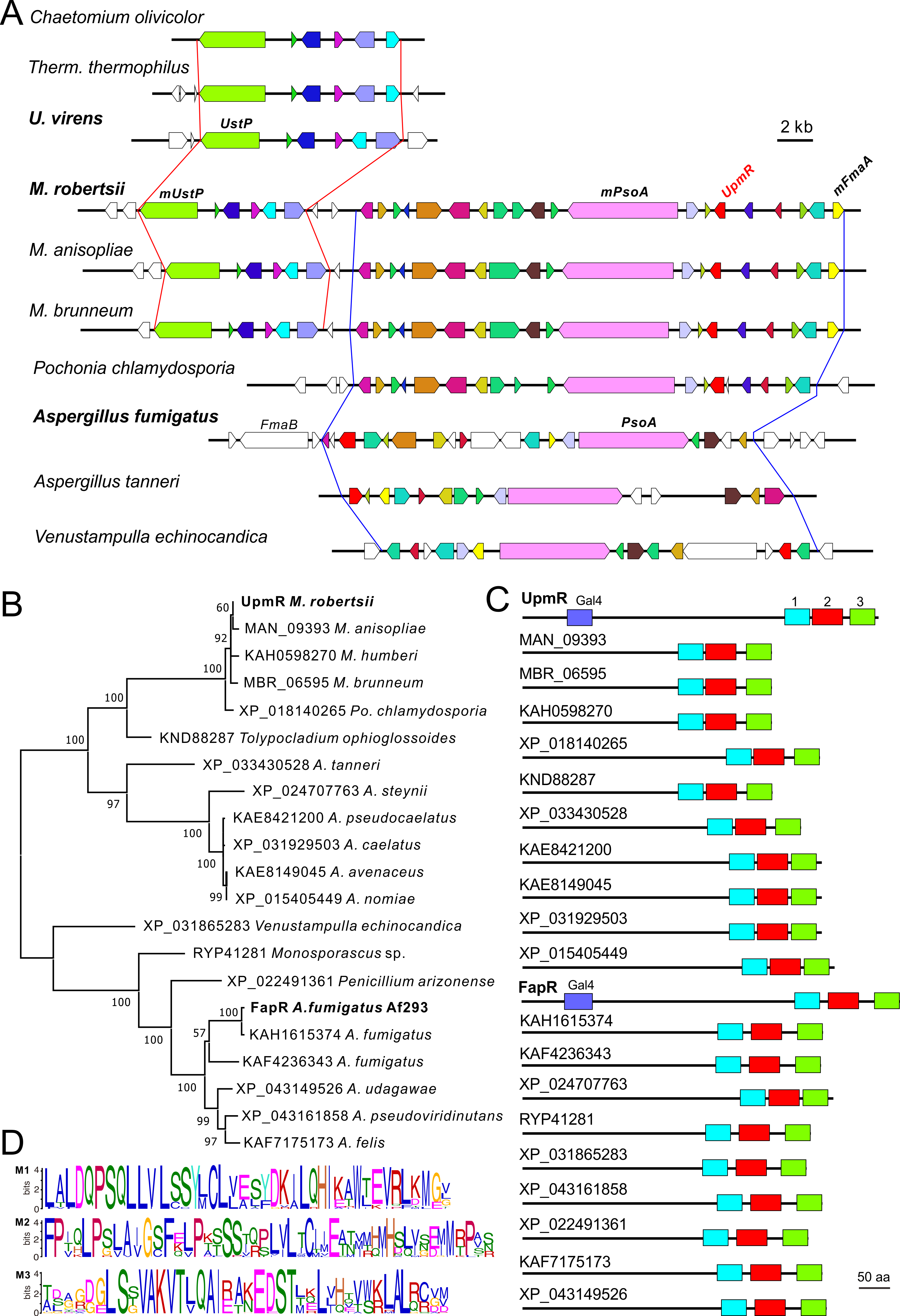

Supplement: FIG S1 [file mbio.01800-22-s0001.tif]

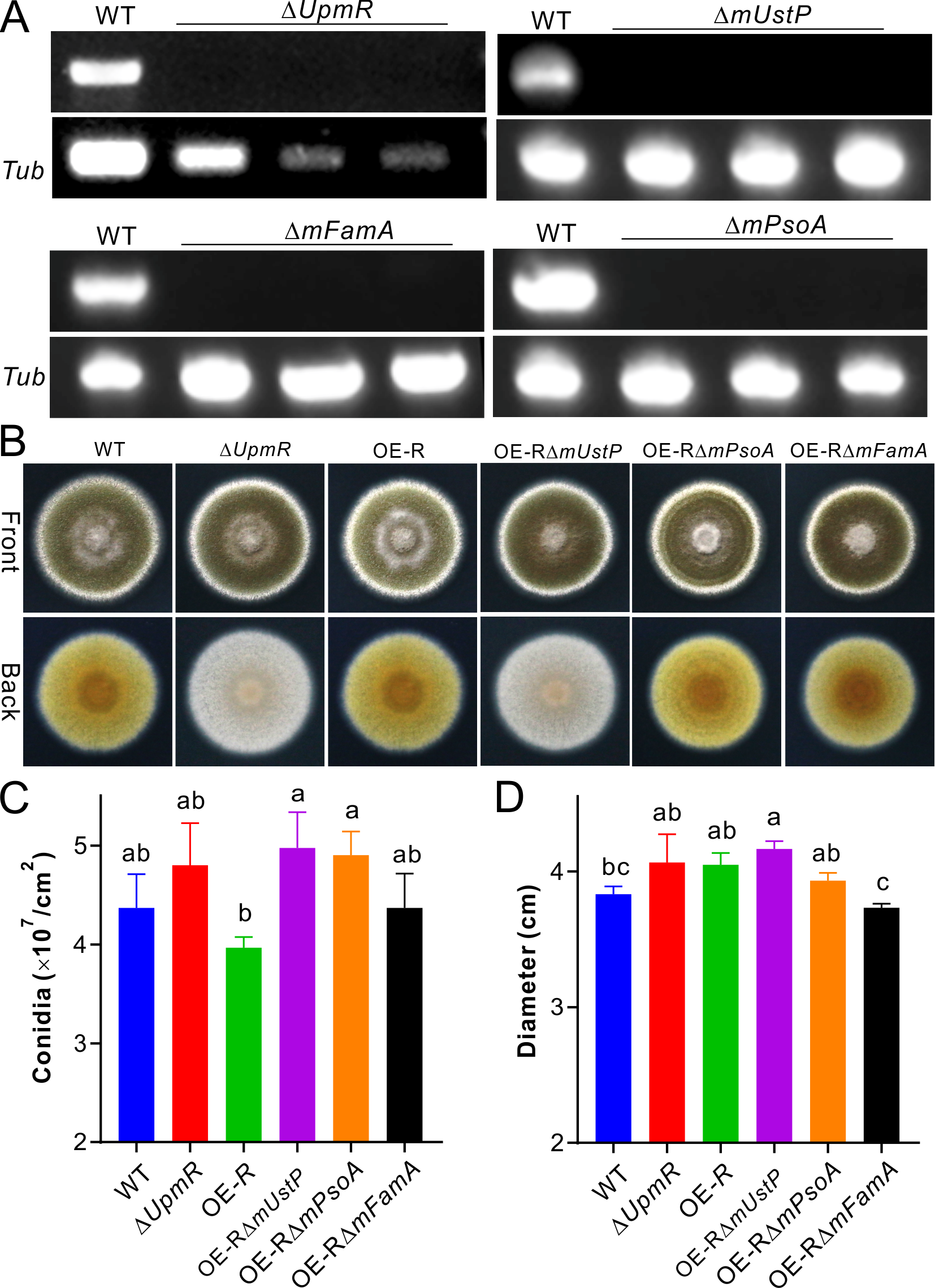

Supplement: FIG S2 [file mbio.01800-22-s0002.tif]

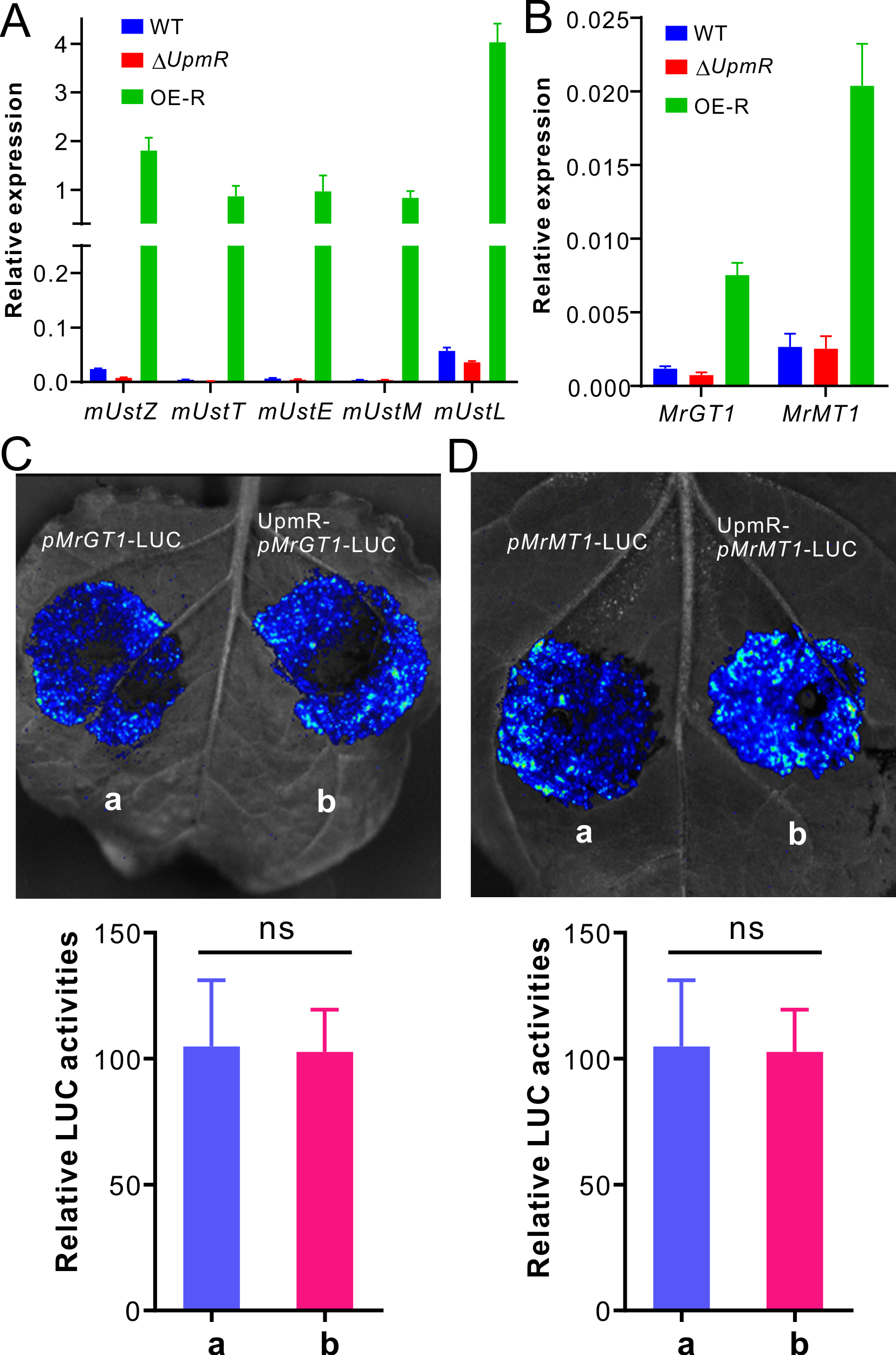

Supplement: FIG S3 [file mbio.01800-22-s0003.tif]

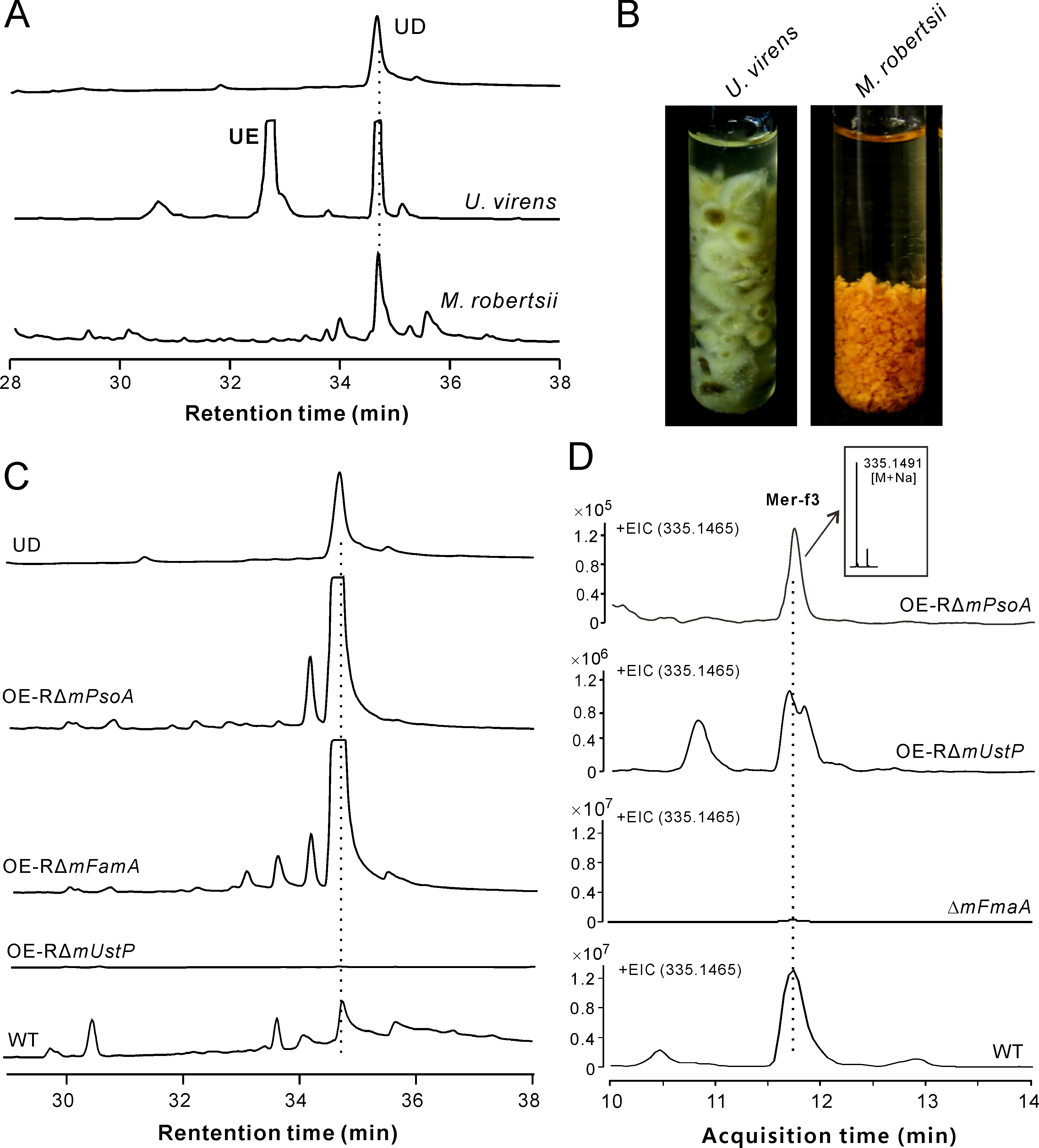

Supplement: FIG S4 [file mbio.01800-22-s0004.tif]

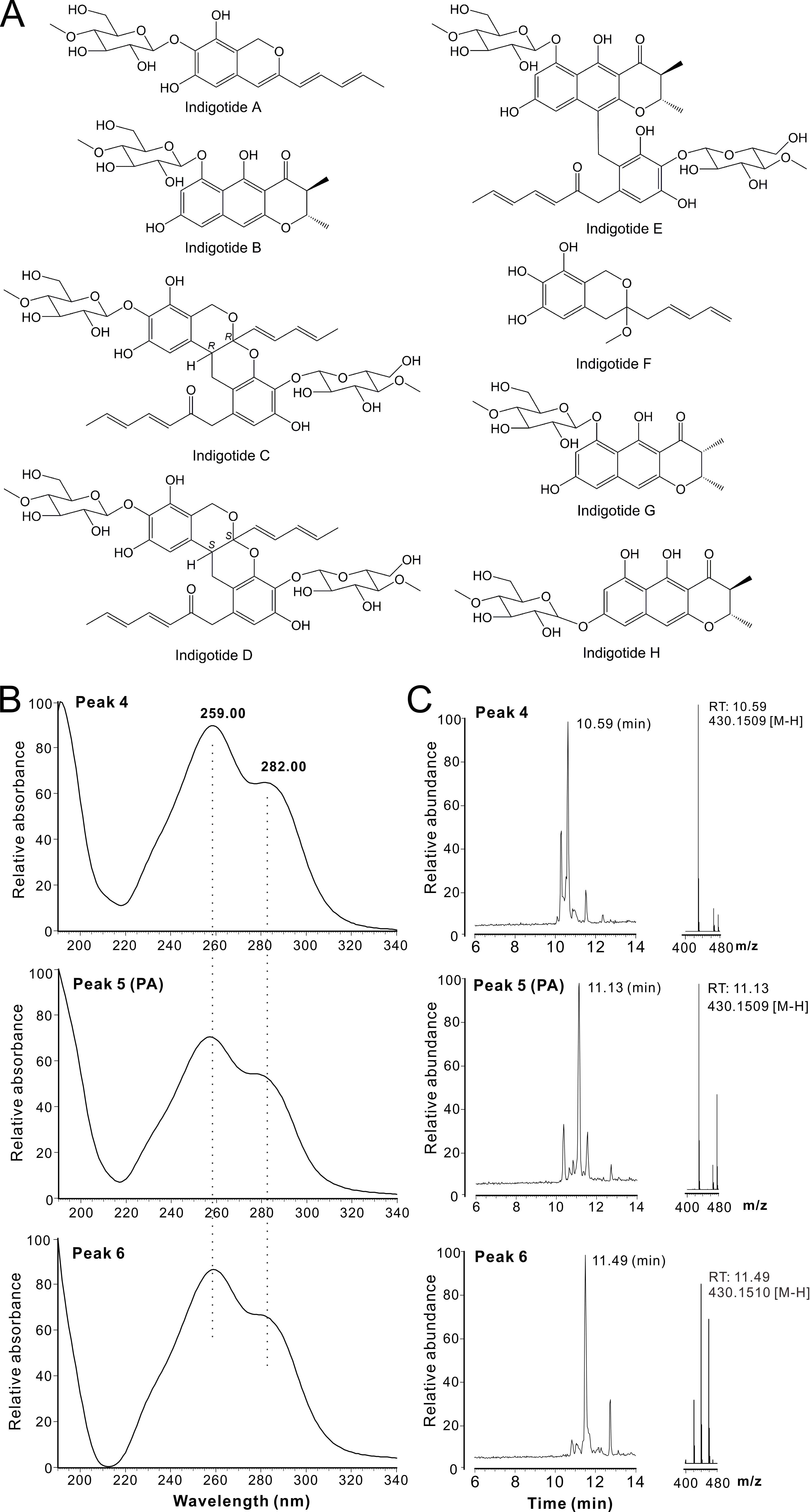

Supplement: FIG S5 [file mbio.01800-22-s0005.tif]

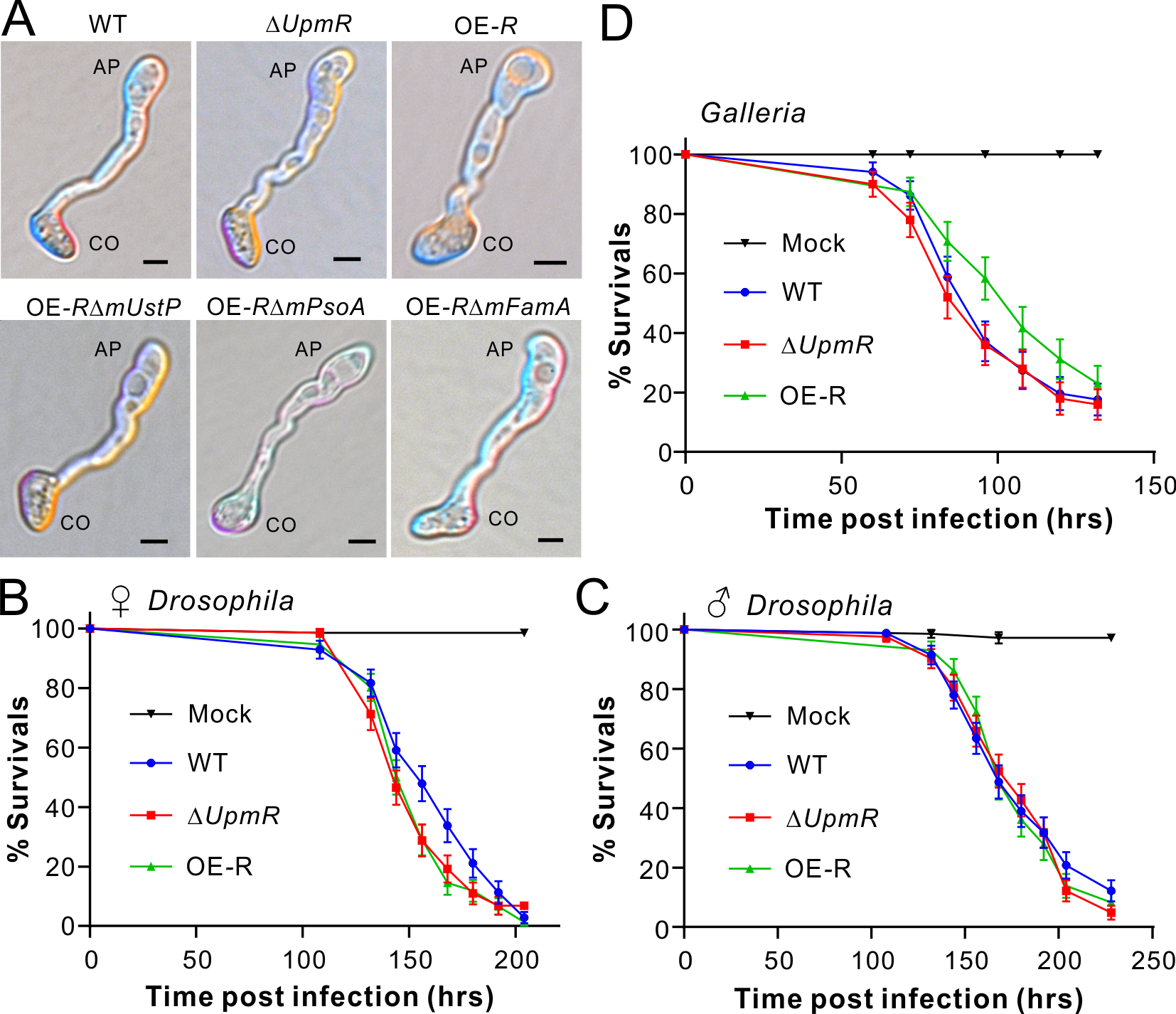

Supplement: FIG S6 [file mbio.01800-22-s0006.tif]

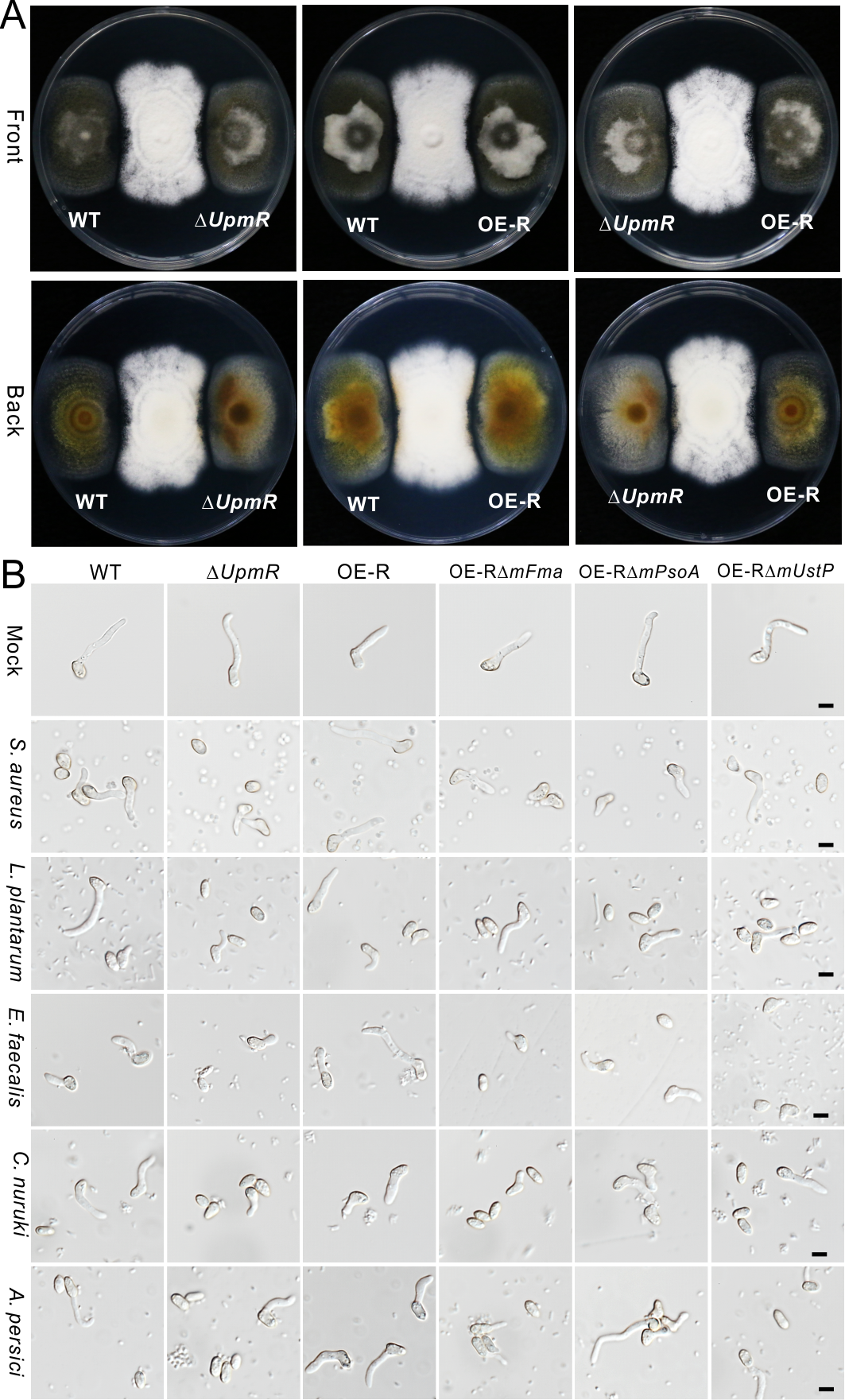

Supplement: FIG S7 [file mbio.01800-22-s0007.tif]
