# Supplementary material for: Orchestrated Biosynthesis of the Secondary Metabolite Cocktails Enables the Producing Fungus to Combat Diverse Bacteria
Source: mBio. 2022 Aug 24;13(5):e01800-22. doi: 10.1128/mbio.01800-22 (PMC9600275; doi:10.1128/mbio.01800-22)
Supplement: DATA SET S1 [file mbio.01800-22-s0009.pdf]

Data set S1: 1D and or 2D NMR spectra of the compounds identified in this study.

^{13}C (125 MHz) and ^{1}H (500 MHz) NMR spectrum data of ustilaginoidin D (in pyridine-*d*5).

Positions	Ustilaginoidin D	
	δ_C, mult.	δ_H (*J* in Hz)
2	79.0, CH	4.17, m
3	46.7, CH	2.79, m
4	201.7, qC	-
4a	102.4, qC	-
5	165.3, qC	15.56, s
5a	105.7, qC	-
6	160.8, qC	9.62, s
7	100.9, CH	6.45, s
8	161.7, qC	8.48, s
9	106.5, qC	-
9a	143.3, qC	-
10	100.0, CH	5.92, s
10a	156.5, qC	-
11	19.9, CH_3	1.39, d, (6.0)
12	10.1, CH_3	1.22, d, (6.0)

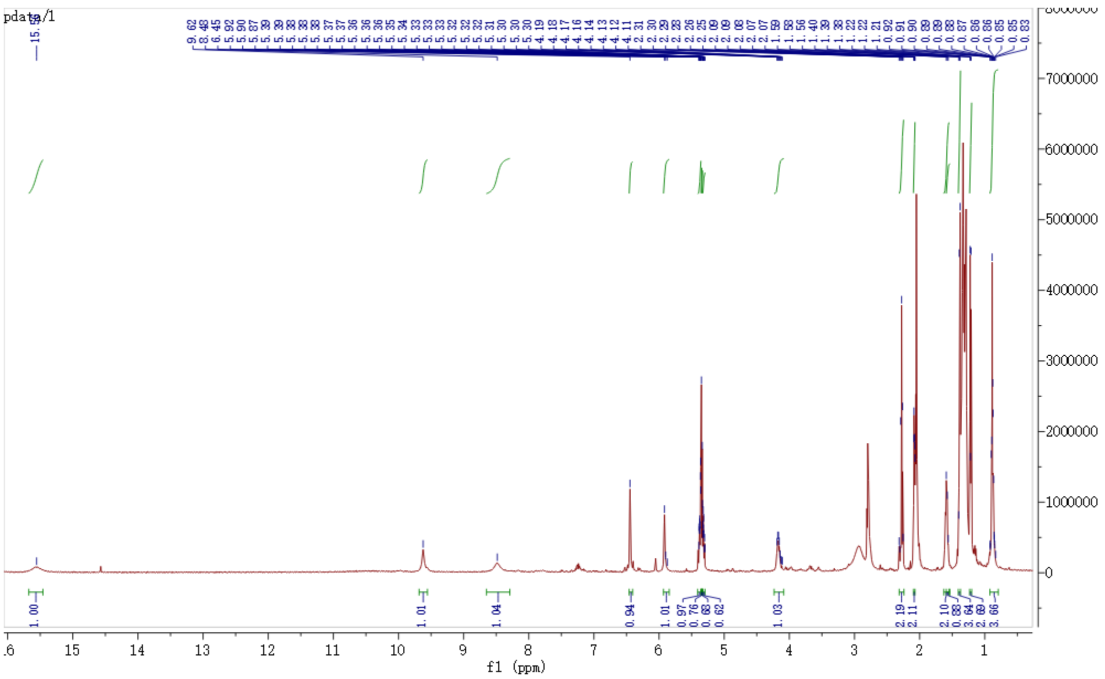

[1] H (500 MHz) NMR data of ustilaginoidin D (in pyridine-*d*5);

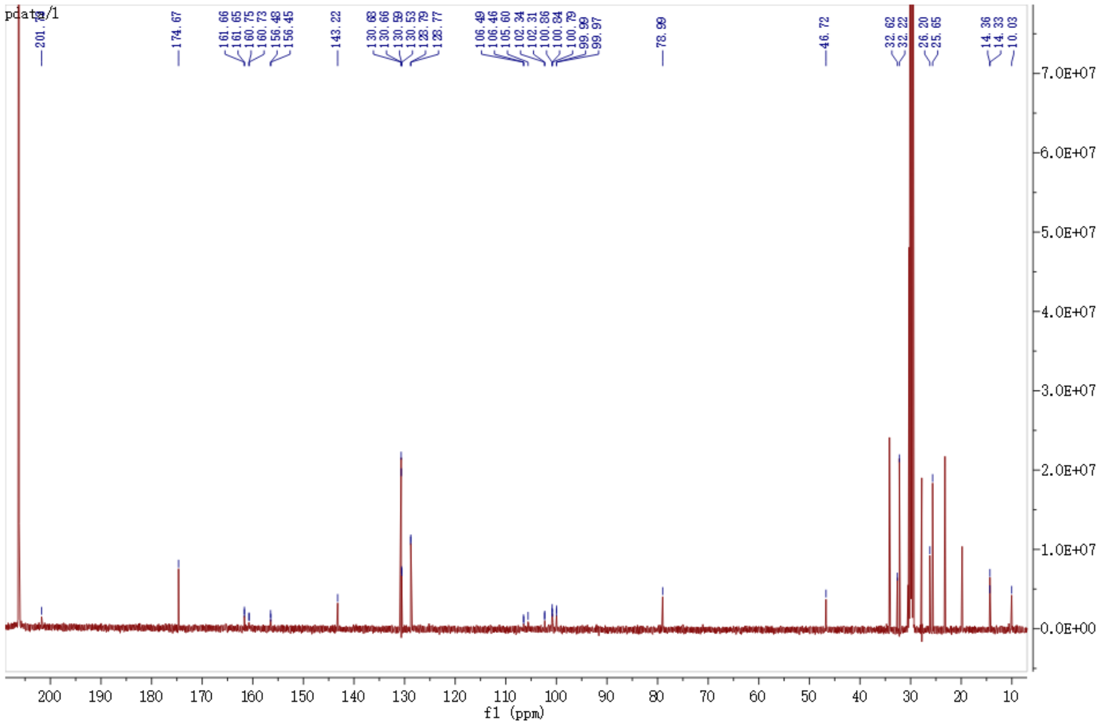

[13] C (125 MHz) NMR data of ustilaginoidin D (in pyridine-*d*5);

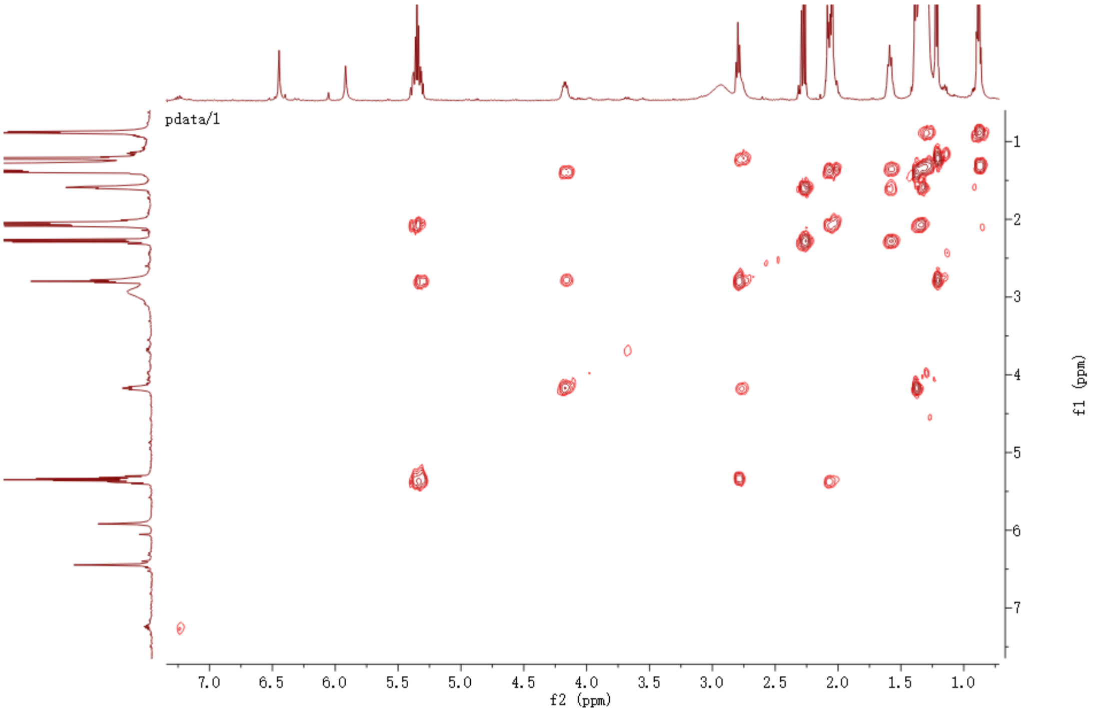

^{1}H-^{1}H COSY (500 MHz) NMR data of ustilaginoidin D (in pyridine-*d5*);

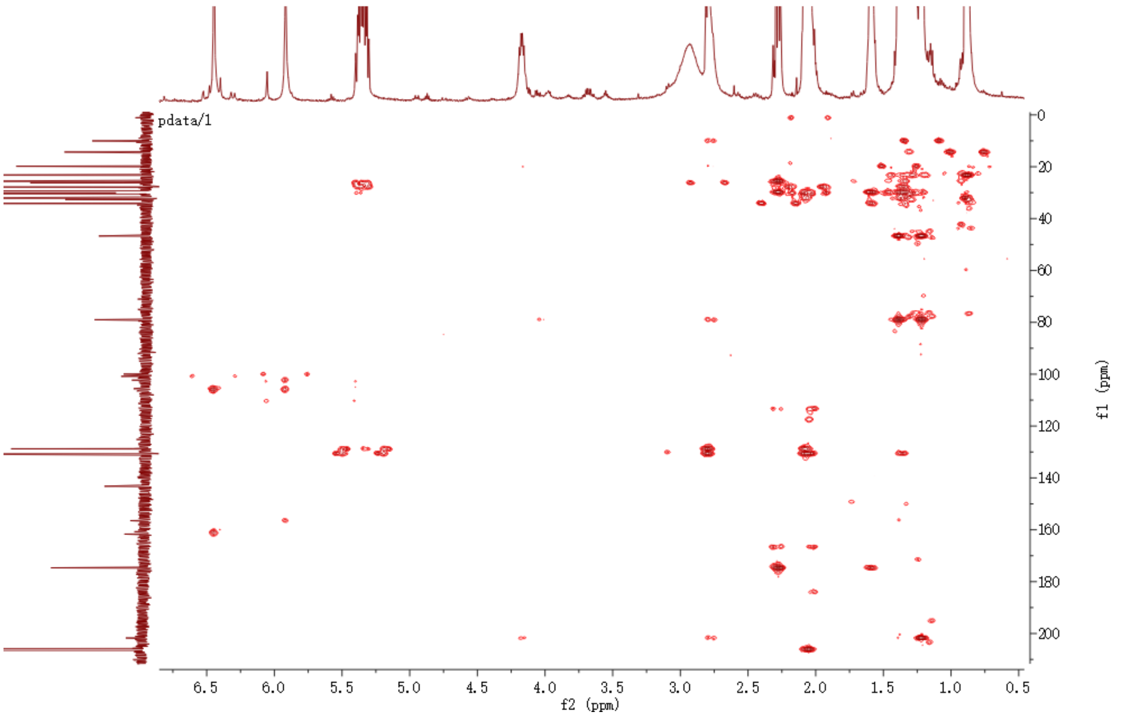

HMBC NMR data of ustilaginoidin D (in pyridine-*d5*);

^{13}C (125 MHz) and ^{1}H (500 MHz) NMR spectrum data of indigotides I, B and J (in pyridine-*d*5).

Positions	Indigotide I		Indigotide B		Indigotide J	
	δ_C, mult.	δ_H (*J* in Hz)	δ_C, mult.	δ_H (*J* in Hz)	δ_C, mult.	δ_H (*J* in Hz)
2	163.7, qC	-	78.1, CH	4.10	165.4, qC	-
3	113.2, qC	-	46.6, CH	2.64, m	112.8, qC	-
4	183.9, qC	-	200.5, qC		183.6, qC	-
4a	103.9, qC	-	103.2, qC		103.1, qC	-
5	163.3, qC	-	166.1, qC		162.9, qC	-
5a	108.2, qC	-	107.1, qC		107.6, qC	-
6	159.8, qC	-	160.6, qC		159.6, qC	-
7	102.9, CH	7.42, d, (2.1)	101.9, CH	7.32, d, (2.1)	101.4, CH	6.98, d, (2.0)
8	161.4, qC	-	162.6, qC		161.2, qC	-
9	103.7, CH	7.14, d, (2.1)	104.3, CH	7.03, d, (2.1)	102.1, CH	7.32, d, (1.9)
9a	141.8, qC	-	144.0, qC		140.7, qC	-
10	100.1, CH	7.04, s	100.9, CH	6.71, s	101.0, CH	7.08, s
10a	153.0, qC	-	156.1, qC		152.4, qC	-
11	18.3, CH_3	2.10, s	19.7, CH_3	1.36	18.5, CH_3	2.16, s
12	9.1, CH_3	1.88, s	10.5, CH_3	1.16, d, (7.0)	8.8, CH_3	1.90, s
1'	103.2, CH	5.62, d, (7.7)	102.9, CH	5.61, d, (7.7)	101.4, CH	5.76, d, (7.7)
2'	75.3, CH	4.44, t, (8.5)	75.2, CH	4.42, t, (8.4)	75.0, CH	4.31
3'	77.7, CH	4.33, t, (9.0)	77.7, CH	4.33, t, (9.0)	78.0, CH	4.38
4'	80.1, CH	3.93, t, (9.2)	80.1, CH	3.92, t, (9.2)	80.4, CH	3.91
4'-OMe	60.7, CH_3	3.85, s	60.6, CH_3	3.86, s	60.6, CH_3	3.89, s
5'	77.7, CH	3.79, m	77.6, CH	3.80, m	77.7, CH	3.98, m
6'	61.6, CH_2	4.13, d, (2.3)	61.6, CH	4.14, d, (3.0)	61.7, CH_2	4.35
-	-	-	-	-	-	4.22, dd, (12.3, 4.5)

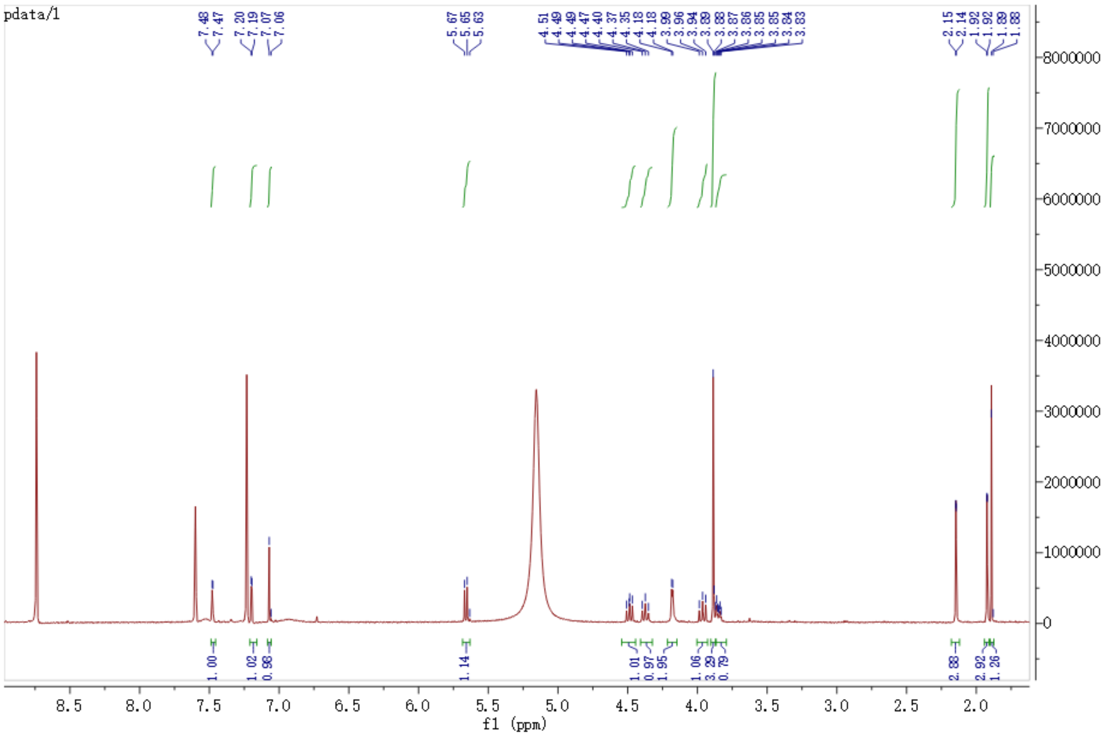

^{1}H NMR spectrum of indigotide I (500 Hz, pyridine-*d*5);

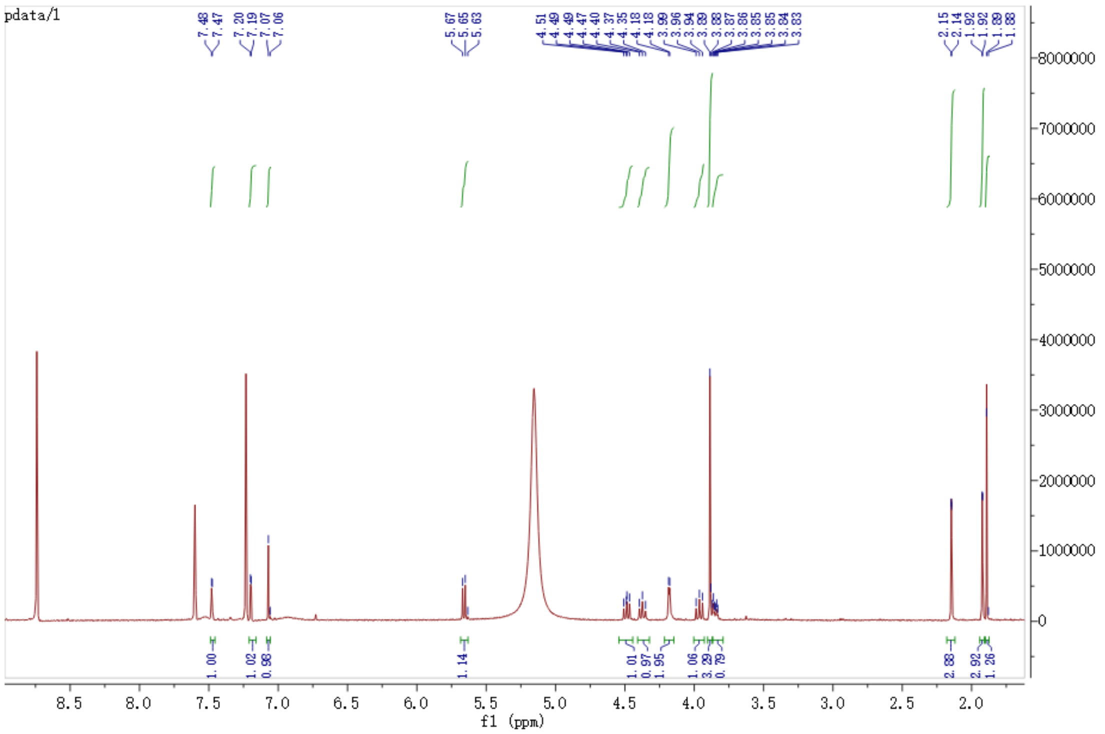

^{13}C NMR spectrum of indigotide I (500 Hz, pyridine-*d*5);

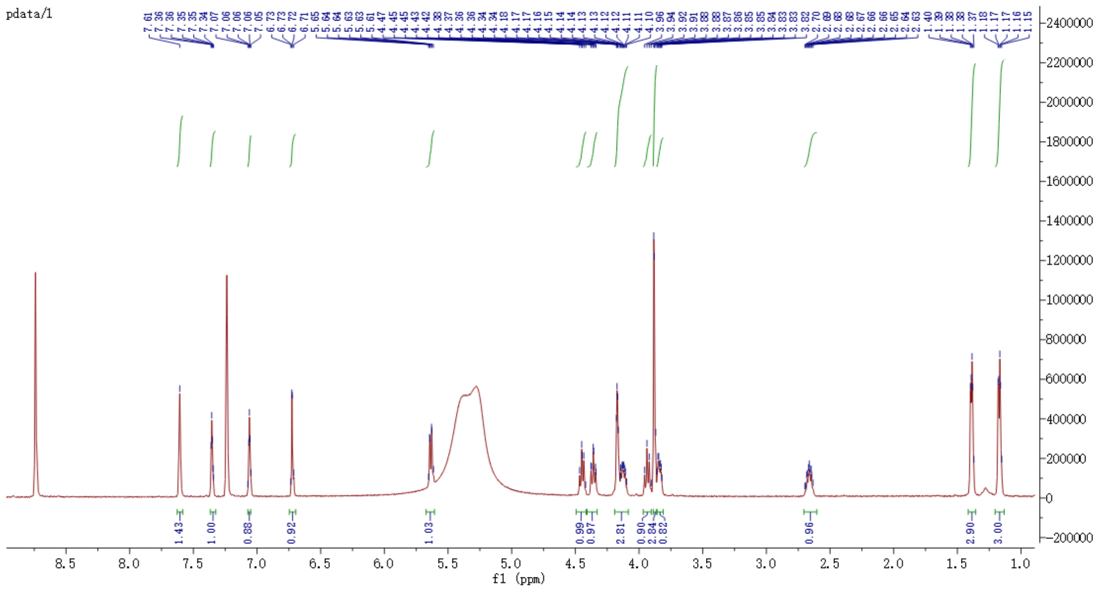

^{1}H NMR spectrum of indigotide J (500 Hz, pyridine-*d*5);

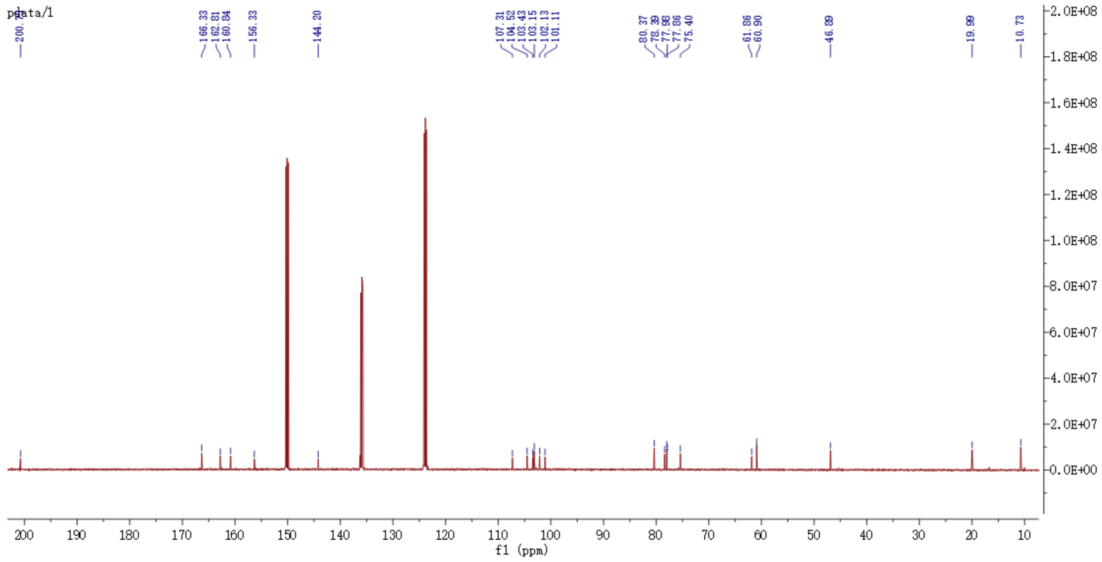

^{13}C NMR spectrum of indigotide J (500 Hz, pyridine-*d*5);

^{13}C (100 MHz) and ^{1}H (400 MHz) NMR spectrum data of Pseurotin A (in CD_3OD).

Positions	Pseurotin A	
	δH, mult (*J* in Hz)	δC
2	-	169.2
3	-	114.4
4	-	199.2
5	-	93.8
6	-	188.7
7	-	
8	-	93.6
9	4.55, s	76.3
10	4.69, d (6.8)	72.9
11	4.52, d (6.8)	69.6
12	5.48, m	128.8
13	5.63, m	137.3
14	2.16, m	22.2
15	0.99, t (7.6)	14.5
16	1.78, s	5.8
17	-	197.1
18	-	134.9
19/23	8.37, dd (8.4, 1.2)	131.7
20/22	7.52, t (8.0)	129.5
21	7.66, t (7.6)	135.1
8-CH_3	3.36, s	52.5

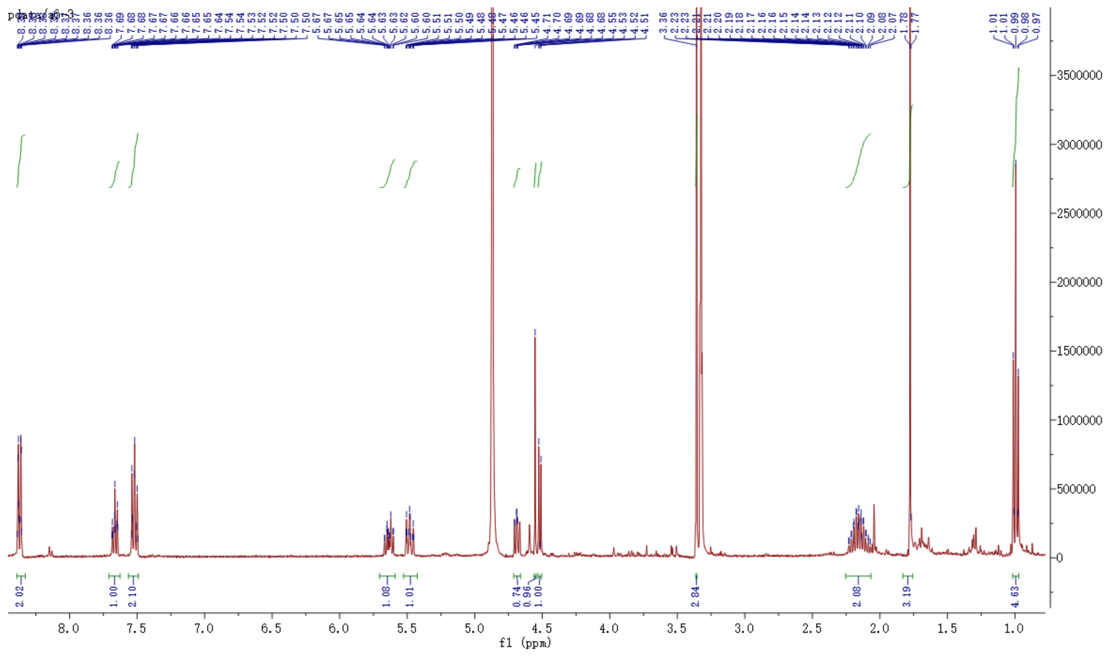

^{1}H NMR spectrum of pseurotin A (400 Hz, CD3OD-d_4);

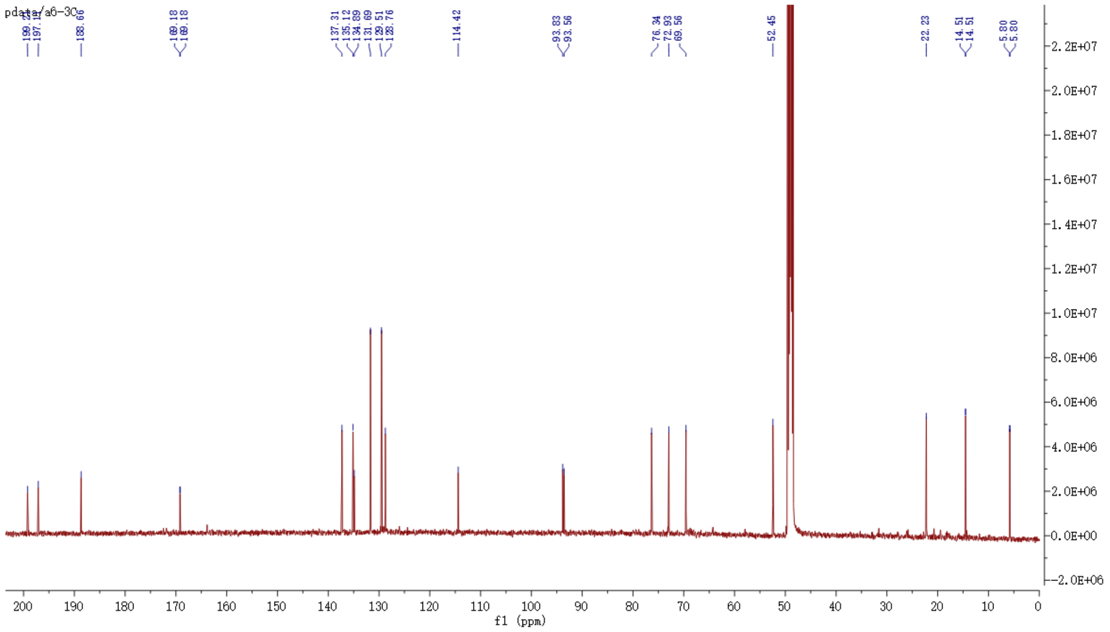

^{13}C NMR spectrum of pseurotin A (100 Hz, CD3OD-d_4);
